# The impact of interventions on appointment and clinical outcomes for individuals with diabetes: a systematic review

**DOI:** 10.1186/s12913-015-0938-5

**Published:** 2015-09-02

**Authors:** Lynn Nuti, Ayten Turkcan, Mark A. Lawley, Lingsong Zhang, Laura Sands, Sara McComb

**Affiliations:** 1 Internal Medicine, Harvard Vanguard, Atrius Health, Boston, MA 02215 USA; 2 Department of Mechanical and Industrial Engineering, Northeastern University, 360 Huntington Avenue, 334 Snell Engineering, Boston, MA 02115 USA; 3 Department of Industrial and Systems Engineering, Department of Biomedical Engineering, Texas A&M University, College Station, TX 77843 USA; 4 Department of Statistics and Regenstrief Center for Healthcare Engineering, Purdue University, West Lafayette, IN 47907 USA; 5 Center for Gerontology, Virginia Tech, Blacksburg, VA 24061 USA; 6 Schools of Nursing and Industrial Engineering, Purdue University, West Lafayette, IN 47907 USA

**Keywords:** Diabetes, Interventions, Clinical outcomes, Behavioral outcomes

## Abstract

**Background:**

Successful diabetes disease management involves routine medical care with individualized patient goals, self-management education and on-going support to reduce complications. Without interventions that facilitate patient scheduling, improve attendance to provider appointments and provide patient information to provider and care team, preventive services cannot begin. This review examines interventions based upon three focus areas: 1) scheduling the patient with their provider; 2) getting the patient to their appointment, and; 3) having patient information integral to their diabetes care available to the provider. This study identifies interventions that improve appointment management and preparation as well as patient clinical and behavioral outcomes.

**Methods:**

A systematic review of the literature was performed using MEDLINE, CINAHL and the Cochrane library. Only articles in English and peer-reviewed articles were chosen. A total of 77 articles were identified that matched the three focus areas of the literature review: 1) on the schedule, 2) to the visit, and 3) patient information. These focus areas were utilized to analyze the literature to determine intervention trends and identify those with improved diabetes clinical and behavioral outcomes.

**Results:**

The articles included in this review were published between 1987 and 2013, with 46 of them published after 2006. Forty-two studies considered only Type 2 diabetes, 4 studies considered only Type 1 diabetes, 15 studies considered both Type 1 and Type 2 diabetes, and 16 studies did not mention the diabetes type. Thirty-five of the 77 studies in the review were randomized controlled studies. Interventions that facilitated scheduling patients involved phone reminders, letter reminders, scheduling when necessary while monitoring patients, and open access scheduling. Interventions used to improve attendance were letter reminders, phone reminders, short message service (SMS) reminders, and financial incentives. Interventions that enabled routine exchange of patient information included web-based programs, phone calls, SMS, mail reminders, decision support systems linked to evidence-based treatment guidelines, registries integrated with electronic medical records, and patient health records.

**Conclusions:**

The literature review showed that simple phone and letter reminders for scheduling or prompting of the date and time of an appointment to more complex web-based multidisciplinary programs with patient self-management can have a positive impact on clinical and behavioral outcomes for diabetes patients. Multifaceted interventions aimed at appointment management and preparation during various phases of the medical outpatient care process improves diabetes disease management.

## Background

Diabetes is a complex chronic illness with significant health and financial implications. It has risen to epidemic proportions in the United States affecting approximately 26 million individuals in 2010 [1]. Projections reveal that if the current increase in diabetes incidence persists and diabetes mortality remains relatively low, prevalence will increase from the current level of 8.3 to 33 % of the adult population by 2050 [2]. Estimates indicate that the United States spent $218 billion in costs for pre-diabetes and diabetes care in 2007 [3]. The American Diabetes Association (ADA) and Healthy People 2020 propose guidelines and objectives for effective diabetes care management to reduce the incidence and economic burden of diabetes [4, 5]. These objectives purport routine medical care with goals and treatment plans individualized for each patient, self-management education and on-going support to reduce the risk of diabetic complications [4].

According to ADA guidelines, which may vary from year to year based on evidence, people with diabetes should receive diabetes self-management education (DSME) at the time their diabetes is diagnosed and as needed thereafter. HbA1c test should be performed at least 2 times a year. The fasting lipid profile (total cholesterol, LDL, HDL, triglycerides) should be measured at least annually. A routine urinalysis and microalbuminuria test should be performed annually to assess nephropathy. A comprehensive foot exam should be performed every year to identify risk factors for ulcers and amputations. A dilated eye exam is recommended every year. Flu vaccines should be provided annually to all patients with diabetes. Pneumococcal vaccines are recommended for all patients over 2 years old. Self-monitoring of blood glucose (SMBG) should be performed three or more times a day for patients using multiple insulin injections or insulin pump therapy.

The percentage of United States adults with diabetes who received preventive care practices in 2009–2010 were as follows: ever attended diabetes self-management class, 57.4 %; check HbA1c ≥ 2 times a year, 68.5 %; annual foot exam, 67.5 %; annual eye exam, 62.8 %; annual flu vaccine, 50.1 %, and; daily self-monitor of blood glucose, 63.6 % [6]. Many factors including demographic, psychological, social, disease, treatment, provider, organizational, and care delivery related factors contribute to poor adherence [7]. These low levels of preventive care suggest an opportunity to enhance adherence to guidelines for effective disease management through appointment management and preparation because before diabetes preventive care practices can be instituted, patients must first be scheduled for and attend their provider appointments. Therefore, this study focuses on organizational and care delivery system related factors that relate to appointment management, as well as regular monitoring of relevant patient information integral to disease management.

Routine medical care starts with scheduling the patient with the provider for preventive care services. The patient can be scheduled for the next visit immediately after a provider visit or at a later time when the patient requests an appointment by phone or electronically. Interventions that proactively schedule the patient with their provider are a necessity for timely treatment decisions. Once patients are scheduled for their provider appointments the next step is to ensure that they attend their appointments. Studies show that no-show rates for diabetic patients vary from 4 to 40 % [8]. Literature also indicates that diabetic patients with higher no-show rates have poorer outcomes e.g., higher glycosylated hemoglobin (HbA1c) levels and poorer glycemic control than patients who attend appointments [8]. Without interventions to encourage patients to schedule and attend their provider appointments, other multifactorial interventions to reduce diabetes complications and costs of care cannot be initiated.

Research indicates that diabetes patients actively involved in their self-management experience improved Quality of Life (QOL) and improved HbA1c levels [9, 10]. Currently, most diabetes care is provided in primary care practices. Accomplishing diabetes care objectives during fifteen to twenty minute appointments can be challenging for primary care providers. A provider cannot prepare individualized patient care without important patient information regarding self-monitoring blood glucoses (SMBG), daily diet and nutrition, exercise or physical activity, and medication information and compliance. To aid in the process of effective disease management, patients must take an informed and active role in the process. Interventions that aid the patient in communicating this information to the provider would expedite patient care delivery and allow the provider more time for individualization of the patient’s treatment plan and patient support in self-management.

Literature examining interventions in diabetes care is extensive and offers a wide variability in types of interventions ranging from medication to web-based self-management tools with varying impact on diabetes outcomes. Different from the earlier literature reviews, the purpose of this literature review is to evaluate interventions that apply to appointment management and preparation, and determine their impact on appointment, clinical and behavioral outcomes for diabetic patients. This review examines interventions based upon three focus areas: 1) scheduling the patient with their provider; 2) getting the patient to their appointment, and; 3) having patient information integral to their diabetes care available to the provider. The hypothesis of this study is that interventions, which improve appointment management and preparation, are significantly associated with favorable appointment, clinical and behavioral outcomes.

## Methods

### Data source

This literature review was completed in February 2014. MEDLINE, the PubMed interface, was the primary database utilized. The following combination of MeSH terms was used for the search: “Diabetes Mellitus”[Mesh] AND (“Intervention Studies”[Mesh] OR “Internet”[Mesh] OR “Reminder Systems”[Mesh] OR “Appointments and Schedules”[Mesh] OR “Patient-Centered Care”[Mesh] OR “Registries”[Mesh] OR “Guideline Adherence”[Mesh]) NOT (“Diabetes, Gestational”[Mesh] OR “Pharmacological Processes”[Mesh] OR “Pharmacological Phenomena”[Mesh] OR “Transplantation”[Mesh] OR “Cardiovascular Surgical Procedures” [Mesh] OR “Heart Diseases”[Mesh] OR “Incidence”[Mesh]). Additionally, the reference lists of included articles and literature reviews were also examined for additional relevant articles. We searched CINAHL and found no additional articles. The Cochrane database was also searched and did not reveal other systematic reviews on this topic.

The search inclusion criteria for the intervention articles were: 1) outpatient diabetes mellitus; 2) adults; and 3) English. The search exclusion criteria eliminated the following types of articles: 1) gestational diabetes; 2) pharmacological processes and phenomena; 3) transplantation (surgery); 4) cardiovascular surgical procedures; 5) heart diseases; and 6) incidence.

### Data extraction

**Fig. 1:**
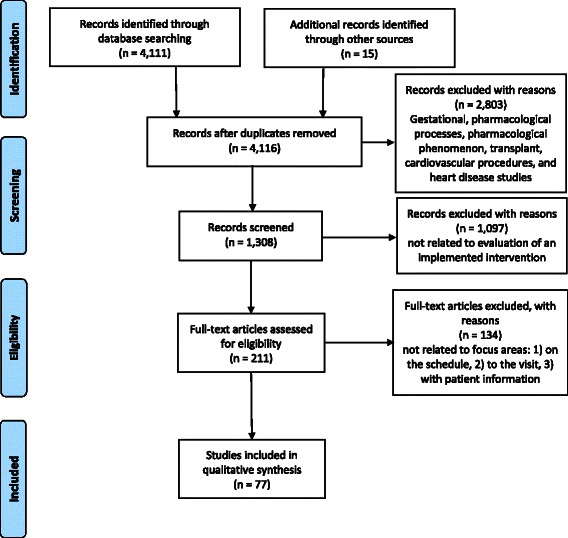
PRISMA flow chart of article selection process

The comprehensive literature search generated 4111 articles (See Fig. 1). Studies excluding gestational, pharmacological process, pharmacological phenomena, transplantation, cardiovascular procedures, heart diseases and incidence reduced potential relevant articles to 2810. Articles were limited to those involving adults (19+ per PubMed), written in English and containing an abstract, which further reduced the total to 1308. Two reviewers reviewed the abstracts independently. All possible articles that could not be excluded were recorded in a table. Each study was marked as “relevant”, “not relevant”, or “maybe” based on the provided information in the paper and the goals for this systematic review. Once the reviewers prepared the tables independently, the decisions were compared and discussed in a meeting. Disagreement regarding inclusion of the article was reconciled through discussion with all other authors. Finally, by excluding articles that were not related to evaluation of an implemented intervention, the sample was reduced to 211 articles. Full texts of the 211 articles were retrieved and outcomes were evaluated independently by two reviewers according to structural, process, and outcomes measures [11]. One hundred and thirty four articles were excluded because they did not relate to the three focus areas: 1) on the schedule, 2) to the visit, and 3) patient information; the remaining 77 articles were included in this literature review. Disagreements regarding interpretation of data extracted from articles were reconciled through discussion with the authors. However, description of the types of interventions and outcomes were summarized and trended.

## Results

The articles included in this review were published between 1987 and 2013, with 46 of them published after 2006. The following is a list of countries and the number of studies from that country included in the review: United States (43); South Korea (15); Netherlands (4); United Kingdom (3); Canada (3); Australia (2); France (1); Finland (1); Iran (1); Italy (1); Norway (1); Taiwan (1) and; Turkey (1). Thirty-five of the 77 studies in the review were randomized controlled studies.

Table 1 is a summary of study designs and interventions used in each article included in this literature review. Appendix 1 provides detailed information about the interventions that focus on three areas of diabetes outpatient care delivery system: 1) scheduling the patient with their provider; 2) getting the patient to their appointment, and; 3) having patient information integral to their diabetes care available to the provider.

**Table 1:** Summary of study designs and interventions

Author		Diabetes type	Study population	Methodology	Intervention
1	Anderson et al. 2003 [15]	98.5 % of intervention group patients are Type 2; 100 % of control group patients are Type 2	*n*_*I*_ = 67, *n*_*C*_ = 65; African Americans; Patients with normal or mild eye exam; Detroit metropolitan area; United States (US).	Randomized Control Trial (RCT); Measurement: 12 months (mos).	Letter and phone reminder
2	Austin and Wolfe 2011 [24]	Not given	*n*_*I*_ = 464, *n*_*C*_ = 693; without HbA1c or LDL-C prior 12 mos; Midwestern university system; US.	Quasi-experimental; Measurement: 12 mos.	Letter reminder with a financial incentive
3	Avdal et al. 2011 [61]	Type 2	*n*_*I*_ = 61, *n*_*C*_ = 61; diagnosis at least 6 mos, > 18 yrs old, on insulin, HbA1c > 7 %, completed diabetes education, can use computer and internet, and volunteered to participate; Turkey.	RCT; Measurements: baseline and 6 mos.	Web-based
			Exclusion: advanced retinopathy or neuropathy.		
4	Bailie et al. 2004 [62]	Type 2	*n*_*B*_ = 137, *n*_*6*_ = 137, *n*_*1*_ = 133, *n*_*2*_ = 123, *n*_*3*_ = 146; Aboriginal people, Australia.	Follow-up study over 3 years; Measurements: baseline, 6 mos, and year 1, 2, and 3.	Electronic Health Record (EHR); Evidence-based Guidelines
5	Benhamou et al. 2007 [63]	Type 1	*n* = 30; ≥ 18 years old, on external insulin pump for 3 mos, and HbA1c 7.5 %-10 %; France.	Bicenter, open-label, randomized, two-period crossover study; 6 mos with SMS (short message service) followed by 6 mos without SMS or reverse sequence; Measurements: baseline and two 6-month periods.	Web-based; SMS
			Exclusion: retinopathy, pregnancy, unable to use software, out of mobile phone network, or unwilling to do 4 SMBG tests/day.		
6	Bond et al. 2006 [64]	Not given	*n* = 15; diabetes, age 60 or older; Washington, US.	Randomized in the first phase, pilot study	Web-based
7	Bond et al. 2007 [36]	87 % Type 1, 13 % Type 2	*n*_*I*_ = 31;*n*_*C*_ = *31;* ≥ 60 years old; having diagnosed with diabetes for at least 1 year, living independently in the community, fluency in English, West coast university health system; US.	RCT; Randomized using two-tier strata (above and below 7.5 % HbA1c) and gender. Intervention subjects participated in one of two phases (each phase lasting one year); Measurements: baseline and 6 mos.	Web-based; Behavioral
			Exclusion: mod/severe cognitive, visual, or physical impairment or severe co-morbid disease.		
8	Carter et al. 2011 [37]	Type 2	*n*_*I*_ = 26, *n*_*C*_ = 21; type 2 diabetes 2 yrs prior to study, ≥ 18 yrs old, African American, 8th grade reading level, residing in Washington, DC, willing provider; US.	RCT; Measurements: baseline and 9 mos.	Web-based; Behavioral
			Exclusion: visually or hearing impaired, non-English speaking, on dialysis or psychotropic meds.		
9	Cavan et al. 2003 [65]	Type 1	*n* = 6; type 1 diabetes and attended one-hour training session; United Kingdom	Pilot study; Measurements: baseline, 3 and 6 mos, and year 1 and 2.	Web-based
10	Cherry et al. 2002 [46]	Not given	*n* = 169; indigent or economically disadvantaged adults, competent, have telephone, can read or have reading assistance, reside and have physician in Mercy Health Center, Laredo, TX service area; US.	Cohort; Measurements: baseline, quarterly for 2 quarters and 12 mos.	Web-based; Telephone data line; Behavioral
11	Cho et al. 2006 [57]	Type 2	*n*_*I*_ = 40, *n*_*C*_ = 40; ≥ 30 yrs old, > 6 mos in center; South Korea.	Prospective, RCT; Measurements: baseline, 3-month intervals up to 30 mos.	Web-based
			Exclusion: disabling conditions, severe diabetes complications, intensified insulin regimen, no internet access, unwilling, or in similar programs.		
12	Cho et al. 2009 [66]	Type 2	Internet: *n* = 37; diabetes phone: *n* = 38; internet access and uses mobile phone/SMS; South Korea.	Randomized, non-inferiority with active-controlled period; Measurements: baseline to 3 mos.	Diabetes Phone; Web-based; SMS
			Exclusion: heart failure, liver enzymes 2x normal, renal disease (creatinine > 1.5 mg/dL), in similar programs.		
13	Cho et al. 2011 [67]	Type 2	*n*_*I*_ = 35, *n*_*c*_ = 36; age ≥ 40; HbA1c from 7.0 to 11.0 %; followed at least 6 months in a public healthcare post in rural areas of Chung-ju, Korea.	RCT; Measurements: Baseline and at 3 months.	Web-based; Phone call; Performance feedback
			Exclusion: diagnosed or suspected disease of liver, pancreas, endocrine organ, kidney; ischemic heart disease; cerebrovascular disease; creatinine >0.133 mmol/l; intensive insulin regimen; unable to attend regularly.		
14	Chumbler et al. 2005 [21]	Not given	*n*_*I*_ = 400, *n*_*C*_ = 400; ≥ 2 Veterans Administration (VA) hospitalizations or emergency visits in last year, telephone access, non-institutionalized; Florida, Puerto Rico and Georgia; US.	Retrospective, concurrent matched cohort; Measurements: 12 mos before and after.	Web-based; Telephone data line
15	Ciemins et al. 2009 [52]	Not given	*n* = 495; adult, provider visit in last year; central/eastern Montana and northern Wyoming; US.	Pre-post intervention, cohort; Measurements: 2 year baseline and two consecutive 2 year intervention periods.	EHR; Registry; Patient and provider report cards; Evidence-based guidelines
			Exclusion: gestational or steroid-induced diabetes, nursing home resident, prednisone use > 2 mos, or seen by endocrinologist for care and testing.		
16	de Grauw et al. 2002 [19]	Type 2	*n* = 432 baseline, *n* = 594 follow-up; type 2 diabetes; Nijmegen Academic Research Network, the Netherlands.	Multicenter cross-sectional; Measurements: baseline and 6 yrs.	Registry; Phone reminder
17	Derose et al. 2009 [25]	Type 1 or 2 (based on ICD-9 codes)	*n*_*I.1*_ = 2916, *n*_*I.2*_ = 1934, *n*_*I.3*_ = 1933, *n*_*I.4*_ = 2199, *n*_*I.5*_ = 2200, *n*_*C*_ = 1875; no HbA1c, LDL-C, and urinary microalbumin tests in > 1 yr, and birthday in 3 mos; Southern California Kaiser Permanent; US.	RCT; Measurements: 2 consecutive 3-month periods.	Letter and phone reminder
18	Dijkstra et al. 2005 [54]	32 % of intervention group patients are Type 1, 33 % of control group patients are Type 1	*n*_*I*_ = 351, *n*_C_ = 418 patients; *n*_*I*_ = 4 *n*_*C*_ = 5 hospitals; *n*_*I*_ = 22, *n*_*C*_ = 20 internists; the Netherlands.	Clustered, RCT; Measurements: baseline and post-intervention (time varied per indicator)	Patient-held record (PHR); Evidence-based guidelines
19	Edelman et al. 2010 [34]	Not given	*n*_*I*_ = 133*, n*_*C*_ = 106; hypertension and diabetes, on diabetes medication, HbA1c > 7.5 % and systolic BP > 140 mm Hg or diastolic BP > 90 mm Hg; North Carolina and Virginia, US.	RCT; Measurements: study midpoint (6.8 mos) and completion (12.8 mos).	Financial incentive; Group visit
			Exclusion: seen by endocrine clinic in past 6 mos, hospitalized for psychosis in past 3 yrs, cognitively impaired, or severe chronic illness.		
20	Edwards et al. 2012 [17]	Type 1 or 2 (based on ICD-9 codes)	*n*_*I*_ = 94, *n*_*C*_ = 210; age 18 and 85 yrs; diabetes patients who were scheduled for appointments with a primary care provider between 08/2010 and 04/2011; University of Oklahoma Family Medicine Center (FMC) in Oklahoma City, US.	RCT; Measurements: 1 year before the intervention, and immediate at intervention	Phone call; Evidence-based guidelines
			Exclusion: pregnant; recently seen in group visits; diabetes managed by a provider outside the FMC.		
21	Farmer et al. 2005 [68]	Type 1	*n*_*I*_ = 47, *n*_*C*_ = 46; United Kingdom; age 18–30 yrs, basal bolus insulin, last 2 HbA1c tests 8 -11 %.	RCT, parallel-group; Measurements: baseline, 4 and 9 mos.	Web-based; SMS
			Exclusion: avoid tight glycemic control, another severe disease, cannot do SMBG, or other family member in trial.		
22	Fischer et al. 2011 [13]	Type 1 or 2 (based on ICD-9 codes)	Mailed report cards: *n*_*I*_ = 2728, *n*_*C*_ = 2729; Printable report cards: *n*_*I*_ = 2357, *n*_*C*_ = 3100; Provider report cards: *n*_*I*_ = 2893, *n*_*C*_ = 2564; >17 yrs, at least one visit to clinic within 18 mos; Denver, CO; US.	Nested randomized trial; Measurements: baseline and 13 mos.	Registry; Patient and provider report cards; Mail reminder
			Exclusion: >75 yrs, no mail address, cannot speak English or Spanish		
23	Fischer et al. 2012 [69]	Not given	*n* = 47; age ≥ 18 yrs; diabetes, have cell phone; fluent in English or Spanish; regularly receive healthcare at a federally qualified community health center in Denver, Colorado, US.	Quasi-experimental; Measurement: at 3 mos.	SMS; Phone call; Behavioral
24	Glasgow et al. 2003 [70]	Type 2	*n* = 320; live by self for ≥ 1 yr; have phone; read and write English; diabetes for at least 1 yr and not moving out of area next yr; Kaiser Colorado, US.	RCT; 3 intervention groups: basic information, tailored self-management and peer support. Measurements: baseline and 10 mos.	Web-based; Behavioral
25	Glasgow et al. 2004 [58]	Type 2	*n*_*I*_ = 469, *n*_*C*_ = 417 patients; *n*_*I*_ = 24, *n*_*C*_ = 28 physicians (all physicians in Diabetes Priority Program); type 2 diabetes, ≥ 25 yrs old, can read English; Colorado; US.	Two-group cluster, RCT; Measurements: baseline and 6 mos.	Web-based
26	Grant et al. 2008 [55]	Type 2	*n*_*I*_ = 126 *n*_*C*_ = 118 patients, *n* = 11 practices; HbA1c > 7 % in prior yr, active diabetes prescription, ≥ 1 visit within prior yr, active account with patient web-portal; eastern Massachusetts; US.	RCT; Measurements: baseline and 12 mos.	Web-based
27	Harno et al. 2006 [71]	Type 1 or 2	*n*_*I*_ = 101, *n*_*C*_ = 74; type 1 or type 2 diabetes; 2 university hospital outpatient clinics; Finland.	RCT; Measurements: baseline and 12 mos.	Web-based; SMS
28	Holbrook et al. 2009 [28]	Type 2	*n*_*I*_ = 253, *n*_*C*_ = 258; ≥ 18 yrs old, fluent in English and able to understand the study description; Ontario, Canada.	Pragmatic RCT; Measurements: baseline and 6 mos.	Web-based; Phone reminder, Behavioral
29	Hurwitz et al. 1993 [72]	Type 2	*n =* 187; non-insulin dependent diabetes mellitus, ≤ 80 yrs old, attend clinic ≥ 2 yrs; United Kingdom.	RCT; Measurements: baseline and 2 yrs.	Letter and phone reminder
			Exclusion: women of childbearing age or patients with significant diabetic complications.		
30	Jones and Curry 2006 [50]	Type 2	*n*_*I*_ = 58, *n*_*C*_ = 115; 2 provider visits during study, and ≤ 1 provider visit in opposite group; Pennsylvania; US	Non-randomized clinical trial; historical control group; Measurements: baseline and within 16 mos after intervention.	Personal digital assistant; Provider reminder; Letter reminder; Evidence-based guidelines
31	HS Kim et al. 2005 [44]	Type 2	*n* = 42; able to do SBMG and self-injection of medication, access to web sites and cellular phone; South Korea.	Quasi-experimental, one group, pretest-posttest; Measurements: baseline and 12 weeks.	Web-based; SMS
			Exclusion: severe illness, renal insufficiency (creatinine > 1.5 mg/dL) or on insulin pump.		
32	HS Kim et al. 2006 [42]	Type 2	*n* = 33; ≥ 30 yrs old, can do SMBG tests and medication injection, can input data to web, internet access, and cellular phone; South Korea.	Quasi-experimental, one group, pretest-posttest; Measurements: baseline and 12 weeks.	Web-based; SMS
			Exclusion: heart failure, hepatic dysfunction, renal insufficiency, on insulin pump or other diabetes web offer.		
33, 34	HS Kim 2007 [39, 40]	Type 2	*n*_*I*_ = 25, *n*_*C*_ = 26; able to do SBMG and self-injection of medication, access to web sites and cellular phone; South Korea.	Control group, pretest-posttest, randomized by random permuted block design; Measurements: baseline, and 3 mos.	Web-based; SMS; Behavioral
			Exclusion: severe illness, renal insufficiency, or on insulin pump.		
35	HS Kim and Jeong 2007 [41]	Type 2	*n*_*I*_ = 25, *n*_*C*_ = 26; able to do SBMG and self-injection of medication, able to input data to web site, had home internet access, and cellular phone; South Korea.	Control group, pretest-posttest, randomized by random permuted block design; Measurements: baseline, 3, and 6 mos.	Web-based; SMS
			Exclusion: severe illness, renal insufficiency, or on insulin pump.		
36	HS Kim and Song 2008 [43]	Type 2	*n*_*I*_ = 18, *n*_*C*_ = 16; ≥ 30 yrs old, obese, able to do SBMG and self-medication, able to input data to web site, had home internet access, and cellular phone; South Korea.	Quasi-experimental, repeated measures, pretest-posttest; Measurements: baseline, 3, and 6 mos.	Web-based; SMS
			Exclusion: heart failure, hepatic dysfunction, renal insufficiency, or on insulin pump.		
37	SI Kim and HS Kim 2008 [73]	Type 2	*n*_*I*_ = 18, *n*_*C*_ = 16; able to do SBMG and self-injection of medication, access to web sites and cellular phone; South Korea.	Quasi-experimental, repeated measures, pretest-posttest; Measurements: baseline, 3, 6, 9, and 12 mos.	Web-based; SMS
			Exclusion: severe illness, renal insufficiency, or on insulin pump.		
38	Kirsh et al. 2007 [12]	Type 2	*n*_*I*_ = 44, *n*_*C*_ = 35; one or more of following: A1c > 9 %, SBP >160 mm Hg and LDL-c >130 mg/dl; Veterans Healthcare System; US.	Quasi-experimental, non-randomized concurrent controls; Measurements: baseline and 6 mos	Letter reminder
39	Kwon et al. 2004 [74]	Type 2	*n*_*I*_ = 51, *n*_*C*_ = 50; type 2 diabetes ≥ 1 yr, internet access, ≥ 30 yrs old; South Korea.	RCT; Measurements: baseline and 12 weeks.	Web-based
			Exclusion: significant diseases likely to affect outcome (heart failure, hepatic dysfunction, renal insufficiency or on insulin pump).		
40	Kwon et al. 2004 [45]	16.2 % Type 1, 82.7 % Type 2, 1.1 % secondary diabetes	*n* = 185; diabetes ≥ 1 yr, internet access; South Korea.	Non-randomized cohort; Measurements: baseline and 3 mos.	Web-based; SMS
			Exclusion: significant diseases likely to affect outcome (hepatic or renal failure).		
41	Lafata et al. 2002 [14]	Type 1 or 2 (based on ICD-9 codes)	*n*_*I*_ = 1641, *n*_*C*_ = 1668; in patient registry, ≥ 18 yrs and ≥ 2 diabetes visits or at least 1 pharmacy claim for diabetes drug in last 24 mos; Michigan, US	RCT; Measurements: 6 and 12 mos.	Letter reminder
42	Lin et al. 2007 [29]	Not given	*n*_*I*_ = 33, *n*_C_ = 35; Canadian primary care center.	Historical cohort; Measurements: baseline and 3 years.	Phone reminder; Evidence-based guidelines; Longer appointments
			Exclusion: no family doctor and those without at least 2 diabetic follow-up appointments.		
43	Litzelman et al. 1993 [75]	Type 2	*n*_*I*_ = 191, *n*_*C*_ = 205; non-insulin dependent diabetes, ≥ 2 visits in prior yr, > 40 yrs old, diabetes diagnosis after age 30, 2 yrs with practice, and ideal or heavier than ideal body weight, at risk of lower-extremity amputation; Indianapolis; US.	RCT; Measurements: baseline and 12 mos.	Phone and postcard reminder; Behavioral
			Exclusion: pregnancy, major psychiatric illness, dementia, terminal illness (death in 1 yr), renal failure, bilateral amputations and investigator’s patients.		
44	Lorig et al. 2010 [76]	Type 2	*n*_*I.1*_ = 209, *n*_*I.2*_ = 186, *n*_*I.3*_ = 395, *n*_*C*_ = 238; aged ≥ 18 yrs, not pregnant or in cancer care, physician verified type 2 diabetes diagnosis and access to the Internet. Effort to recruit American Indians/Alaskan Natives; California; US.	RCT; Measurements: baseline, 6, and 18 mos.	Web-based
45	Maclean et al. 2009 [20]	Type 1 or 2	*n*_*I*_ = 3886, *n*_*C*_ = 3526 patients; *n*_*I*_ = 70, *n*_*C*_ = 62 physicians; *n*_*I*_ = 30, *n*_*C*_ = 34 practices; HbA1c in last 2 yrs; Vermont and New York; US.	RCT; Practices randomized in blocks by hospital laboratory; Measurements: 32 mos.	Registry; Decision support; Fax and Letter reminder
			Exclusion: < 18 yrs, cognitive impairment or provider decision.		
46	McCarrier et al. 2009 [77]	Type 1	*n*_*I*_ = 41, *n*_*C*_ = 36; 21–49 yrs old, ≥ 2 encounters and at least 1 HbA1c in prior yr, recent HbA1c >7% and reside in King or Snohomish County, Center, Washington; US.	Randomized, pretest-posttest trial; Measurements: 12 mos.	Web-based
47	McDermott et al. 2001 [32]	Not given	*n* = 282 patients at 8 intervention sites, *n* = 396 patients at 13 control sites; mostly Torres Strait Islanders, Australia	Randomized unblended, cluster trial; Measurements: baseline and 12 mos.	Registry; Evidence based guidelines
48	McDiarmid et al. 2001 [51]	Type 2	*n* = 258; urban family practice residency, Greensboro, North Carolina; US.	Non-randomized, before/after, retrospective chart audit; Measurements: baseline and 12 mos.	Evidence-based guidelines
49	McMahon et al. 2005 [78]	Not given	*n*_*I*_ = 52, *n*_*C*_ = 52; HbA1c ≥ 9 %, age > 18 yrs, understands written and spoken English, willingness to use notebook computer, glucose and BP devices; Boston VA Healthcare System; US.	RCT; Measurements: baseline, 3, 6, 9 and 12 mos.	Web-based
50	McMahon et al. 2012 [47]	Type 2	*n*_*I.1*_ = 51, *n*_*I.2*_ = 51, *n*_*I.3*_ = 49; age > 25 yrs, HbA1c > 8.5 %, understand written and spoken English, access to phone, willingness to use laptop, and BP and glucose monitoring devices, have a VA-based primary care provider; Boston, MA; US.	RCT; Measurements: 3, 6, 9, and 12 mos.	Web-based; Phone calls; Performance feedback
51	Mehler et al. 2005 [79]	Type 2	*n*_*I.1*_ = 415, *n*_*I.2*_ = 146, *n*_*C*_ = 323 patients at 12 primary care practices; age ≥ 40 yrs; Denver-metro area; US.	Stratified and randomized by practice type (family medicine, internal medicine or academic); Measurements: baseline and 15 mos.	Evidence-based guidelines
52	Meigs et al. 2003 [49]	Type 2	*n*_*I*_ = 307 patients, *n*_*I*_ = 12 providers; *n*_*C*_ = 291 patients, *n*_*C*_ = 14 providers; hospital-based staff-resident medical practice; Boston, Massachusetts; US.	RCT; Measurements: 12 mos pre-intervention and 12 mos post-intervention.	Web-based; Decision support; Evidence-based guidelines
53	Meulepas et al. 2007 [30]	Type 2	*n*_*I*_ = 353 patients, *n*_*I*_ = 51 providers; *n*_*C*_ = 129 patients, *n*_*C*_ = 27 providers; documented diabetes for > 4 yrs at start of study; The Netherlands	Controlled, non-randomized, before/after study with delayed intervention in control group; Measurements: 1 yr before intervention and 2 years after.	Phone reminder
54	Meulepas et al. 2008 [31]	Type 2	*n*_*I*_ = 431 patients, *n*_*I*_ = 23 providers; *n*_*C*_ = 469 patients, *n*_*C*_ = 28 providers; in the south of The Netherlands	Controlled, non-randomized study, before/after; Measurements: 1 yr before intervention and 2 years after.	Phone reminder
55	Moattari et al. 2013 [80]	97 % Type 1	*n*_*I*_ = 24, *n*_*C*_ = 24; have diabetes, need insulin, ability to use glucometer and inject insulin, ability to input data on a website, own cellphone; Shiraz, Iran.	RCT; Measurements: baseline and 3 mos.	Web-based; Phone; SMS
			Exclusion: chronic disease or renal failure (creatinine > 1.5 mg/dl), use of insulin pump, pregnancy.		
56	Moorman et al. 2012 [81]	Not given	*n*_*C*_ = 19*, n*_*I*_ = 18*;* Adult diabetic patients not working with a case manager, at least one request for a self-monitoring blood glucose log, Ohio, US	Cohort study; Measurements: 3 mos. before the intervention and 3 mos. after.	Letter reminder
			Exclusion: No documented mailing address		
57	Musacchio et al. 2011 [82]	Type 2	*n =* 1004; HbA1c < 7 %, ability to follow educational program, and clinical data for prior 12 mos; Italy.	Pre-post study; Measurements: baseline and 12 mos.	Tele-medicine (phone and internet); EHR; Behavioral
58	Nes et al. 2012 [83]	Type 2	*n* = 11; type 2 diabetes, no other inclusion/exclusion criteria reported; Oslo, Norway	Snowball sample pilot study; baseline and 3 mos.	Web-based; Performance feedback
59	Piette et al. 2000 [84]	Not given	n = 248; English or Spanish speaking adults; California; US.	Randomized control trial; Measurements: baseline and 12 mos.	Automated phone call
			Exclusion: >75 yrs, psychotic, sensory impairment, or life expectancy <12 mos, on hypoglycemic medication, diabetes ≤ 6 mos, plan to stop clinic services during study period, no push-button phone.		
60	Rai et al. 2011 [18]	Type 1 or 2 (based on ICD-9 codes)	*n*_*I*_ = 1765, *n*_*C*_ = 1315; 2 diabetes and hypertension ICD-9 codes in billing data in past 2 yrs; no provider visit in last 6 mos; Wisconsin; US.	Quasi-experimental; Measurement: 6 mos.	Phone reminder
			Exclusion: patient without history of treatment by provider.		
61	Ralston et al. 2009 [38]	Type 2	*n*_*I*_ = 39, *n*_*C*_ = 35; 18–75 yrs old, last HbA1c ≥ 7 %, at least two visits in prior year; University of Washington; US.	Randomized, single-centered, controlled trial with parallel group design; Measurements: 12 mos before intervention and 12 mos after.	Web-based; Decision support
			Exclusion: in pilot, psychological illness, non-English speaking, resident as provider or mostly specialty care.		
62	Ryan et al. 2013 [85]	Type 2	*n*_*I*_ = 24; age 21 and older; established patient; seen at least once for diabetes management during the previous 12 months; Most recent A1c < 10; last A1c within last 6 months; a community health clinic in Miami, Florida, US.	Pretest-posttest; Measurements: baseline and 13 mos.	Web-based
			Exclusion: Did not speak English; had an emergency room discharge or hospital admission for a diabetes-related complication during the 6 months before recruitment; were homeless or did not have control of the given living situation; had significant cognitive impairment or psychological distress; had known substance or alcohol abuse.		
63	Sacco et al. 2009 [48]	Type 2	*n*_*I*_ = 31, *n*_*C*_ = 31; age18 – 65 yrs, reads and speaks English, reachable by phone, HbA1c > 6.5 %, cardiovascular risk factor; Florida; US	Randomized, pretest-posttest; Measurements: baseline and 6 mos.	Behavioral; Phone coaching
			Exclusion: major medical/mental disorder.		
64	Sadur et al. 1999 [22]	Type 1 or 2	16-75 yrs old, recent HbA1c > 8.5 % or no HbA1c in last year; Kaiser; California; US.	RCT; Measurements: baseline and 6 mos. Hospitalization rate measured 12 mos before intervention and 18 mos after.	Group visit; Phone; Behavioral
			Exclusion: pregnancy, dementia, no English, cannot attend monthly meetings.		
65	Seto et al. 2012 [16]	Type 1 or 2	*n*_*I*_ = 580; age 18 and older; seen at the health center between July 1, 2009 and June 30, 2010; a primary care clinic in San Jose, California, US.	Pretest-posttest; Measurements: baseline and 7 mos.	Registry; Appointment reminder
			Exclusion: No baseline A1c; gestational diabetes		
66	DM Smith et al. 1987 [27]	Not given	*n*_*I*_ = 425, *n*_*C*_ = 429; patients with insulin or oral hypoglycemic agents prescribed, reported all care received at center, not residents of nursing home or other institution, ≥ 15 yrs old, visited clinic in last yr and had scheduled appointment to return to clinic; metropolitan Indianapolis; US.	RCT; Measurement: 2 yrs.	Letter and phone reminder
67	KE Smith et al. 2004 [86]	Type 1 or 2	*n* = 16; ≥ 18 yrs old, no unstable cardiac disease or organ transplantation, can read computer monitor, and HbA1c > 8.5 %; Georgetown University Hospital; US.	Non-randomized, prospective feasibility; Measurements: baseline and 6 mos.	Web-based
68	Song et al. 2009 [87]	Type 2	*n*_*I.1*_ = 15, *n*_*C*_ = 16; adults, new diagnosis type 2 diabetes, never attended formal self-management education by health professional or over internet; Seoul, Korea.	Quasi-experimental, non-equivalent control group, pretest-posttest; Measurements: baseline, 6 weeks, and 3 mos.	Web-based; Behavioral
69	Stone et al. 2012 [88]	Not given	*n*_*I.1*_ = 21, *n*_*I.2*_ = 23, *n*_*I.3*_ = 28, *n*_*I.4*_ = 29; age 18–79 yrs; diagnosis defined as 12 or more months of pharmacologic treatment for diabetes; HbA1c ≥ 7.5 %; no comorbid conditions indicating life expectancy of less than 5 years; private residence with telephone land line; VA Healthcare System, Pittsburgh, Pennsylvania, US.	RCT; pretest-posttest; Measurements: baseline, 3, and 6 mos.	Tele-monitoring (phone); Performance feedback
			Exclusion: Did not have a telephone landline.		
70	Subramanian et al. 2009 [23]	Type 2	*n*_*I*_ = 3147*, n*_*C*_ = 913; prescription refill for hypoglycemic agent without polycystic ovarian disease, HbA1c ≥ 9 % or elevated FBS ≥ 200 mg/dL; Indianapolis; US.	Retrospective, cohort; Measurements: 1 yr before intervention and 1 yr after.	Open access (OA)
			Exclusion: missing all lab tests, vital signs, or visit data in study period.		
71	Tang et al. 2013 [89]	Type 2	*n*_*I*_ = 193, *n*_*C*_ = 189; age ≥ 18 yrs; HbA1c ≥ 7.5 %; seen within the past 12 months; a not-for-profit healthcare organization in Palo Alto, California, US.	RCT; Measurements: Baseline, 6 and 12 mos.	Web-based; Performance feedback; EHR; Behavioral
			Exclusion: initial diagnosis within the last 12 months; inability to speak or read English; lack of regular internet access; unwillingness to perform self-monitoring at home; diagnosis of a terminal disease and/or entry into hospice care; pregnancy, planning pregnancy or currently lactating; enrollment in another care management program; resident of a long-term facility; uninsured; plans to discontinue primary care at current location; family household member enrolled in EMPOWER-D study.		
72	Thomas et al. 2007 [26]	Not given	*n*_*I*_ = 78 resident physicians, *n*_*C*_ = 39; Internal Medicine residents; Mayo Clinic, Minnesota; US.	RCT; Randomization stratified by clinic day across 5 practice sections; Measurements: baseline and completion including prior 6 mos for HbA1c and prior 12 mos for lipids.	Registry; Evidence-based guidelines; Performance feedback; Letter reminder
73	Tildesley et al. 2010 [90]	Type 2	*n*_*I*_ = 24*, n*_*C*_ = 23; on insulin alone or with oral hypo-glycemic medication, recent HbA1c >7.0 %, internet access, and training in SMBG; Vancouver, Canada	RCT; Measurements: baseline, 3 and 6 mos.	Web-based; Performance feedback
74	Weber et al. 2008 [53]	Not given	Gesinger Health System of 38 practice sites and > 20,000 diabetes patients >18 years old in 40-county region of central and northeastern Pennsylvania; US.	Retrospective, cohort; Measurements: baseline time period (12 mos before) and monthly after implementation of intervention for 12 mos.	Registry; Evidence-based guidelines; Provider reminder; Performance feedback
75	Yeh et al. 2006 [33]	Type 2	*n*_*I*_ = 134, *n*_*C*_ = 140; medical teaching hospital in Taipei, Taiwan	RCT; Measurements: pre-intervention and post-intervention (8 month follow-up).	Web-based; SMS;
76	Yoo et al. 2009 [91]	Type 2	*n*_*I*_ = 57, *n*_*C*_ = 54; age 30 and 70 yrs; hypertension and type 2 diabetes diagnoses in last year; HbA1c 6.5–10.0 %; BP > 130⁄80 mmHg; BMI ≥ 23.0 kg⁄m^2^; Seoul, Korea.	RCT; Measurements: base line and 3 mos.	Web-based; Phone reminder; Telephone data line; Automated performance feedback; SMS
			Exclusion: Severe diabetic complications; liver dysfunction with enzymes >2.5x normal, or renal dysfunction, diagnoses of heart failure, angina, myocardial infarction, or stroke, pregnancy or lactation.		
77	Yoon and HS Kim 2008 [92]	Type 2	*n*_*I*_ = 25, *n*_*C*_ = 26; ability to perform SBMG, access websites, and cellular phone with web access; university medical center, urban city of South Korea.	RCT, pretest-posttest; Measurements: baseline, 3, 6, 9, and 12 mos.	Web-based; SMS
			Exclusion: severe illness, renal insufficiency with creatinine > 1.5 mg/dL or on insulin pump.		

*I* intervention group, *C* control group

The reviewed articles evaluated the impact of interventions on several outcome measures. We divided the outcome measures into two types: clinical outcomes and behavioral outcomes. Clinical outcomes include the value of laboratory test results such as HbA1c, LDL, HDL, systolic blood pressure (SBP), diastolic blood pressure (DBP), total cholesterol, triglycerides, fasting plasma glucose, creatinine, 2-hour post meal glucose, and the value of clinical measures such as weight and body mass index (BMI). Given the importance of HbA1c in diabetes care, Table 2 includes only HbA1c results. All other clinical outcomes are provided in Appendix 2. In Table 2 and Appendix 2, we present the difference between the clinical outcome value at baseline and after the intervention (e.g., HbA1c at baseline – HbA1c at *m* months after the intervention) for both intervention and control groups. Where available, the p-values are presented for the difference between groups and the difference within the groups.

**Table 2:** Changes in HbA1c

	Author	On schedule	To visit	With information	HbA1c at baseline		Change in HbA1c		P-value	Comparisons tested
					Intervention group	Control group	Intervention group	Control group		
39	Kirsh et al. 2007 [12]	✓			10.4	9.8	−1.44	0.30	.002	Group × Time interaction @18 mo.
70	Subramanian et al. 2009 [23]	✓			7.7	7.5	−0.19	−0.03	≤0.05	Group × Time interaction @1 year
3	Avdal et al. 2011 [61]			✓	8.0	8.1	−0.5	NA	≤.010	Time effect @6 mo.
							NA	0.05	NS	Time effect @6 mo.
5	Benhamou et al. 2007 [63]			✓	8.3	8.2	−0.14	0.12	.097	Group effect @6 mo.
7	Bond et al. 2007 [36]			✓	7.0	7.1	−0.6	−0.1	0.01	Group effect @6 mo.
8	Carter et al. 2011 [37]			✓	9.0	8.8	−2.18	−0.9	≤.050	Group effect @9 mo.
9	Cavan et al. 2003 [65]			✓	9.7	NA	−1.7^a^	NA	≤.005	Patients with a disease duration ≤ 10 years
										Time effect @2 year
					9.5	NA	−0.3^a^	NA	NS	Patients with a disease duration > 10 years
										Time effect @2 year
12	Cho et al. 2009 [66] (phone)			✓	8.3	NA	−1.1	NA	≤.010	Time effect @3 mo.
	Cho et al. 2009 [66] (internet)			✓	7.6	NA	−0.6	NA	<.010	Time effect @3 mo.
13	Cho et al. 2011 [67]			✓	8.0	8.0	−0.5	−0.2	<0.01	Time effect @3 mo.
18	Dijkstra et al. 2005 [54]			✓	8.1	8.0	−0.3	0.2	≤.001	Group effect @1 year
21	Farmer et al. 2005 [68]			✓	9.2	9.3	−0.6^a^	−0.4^a^	0.33	Group effect @9 mo.
24	Glasgow et al. 2003 [70] (peer support)			✓	7.54	7.35	−0.12	0.33	≤.05	Group × Time interaction @10 mo.
	Glasgow et al. 2003 [70] (tailored self-management)			✓	7.45	7.43	−0.03	0.24	NS	Group × Time interaction @10 mo.
26	Grant et al. 2008 [55]			✓	7.3	7.4	−0.16	−0.26	0.62	Group effect @1 year
27	Harno et al. 2006 [71]			✓	7.82	8.21	−0.50	NA	S	p ≤ .05 Group effect @1 year
							NA	−0.38	S	
33	HS Kim et al. 2006 [42] “Impact of a nurse short message service intervention…”			✓	8.1	NA	−1.10	NA	.006	Time effect @3 mo.
34	HS Kim 2007 [39] “A randomized controlled trial of a nurse short-message…”			✓	8.09	7.59	−1.15	0.07	.005	Group × Time interaction @3 mo.
35	HS Kim 2007 [40] “Impact of web-based nurse’s education…”			✓	6.92	6.71	−0.21	NA	0.20	Patients with a baseline HbA1c < 7 %
										Time effect @3 mo.
							NA	0.43	.034	Patients with a baseline HbA1c < 7 %
										Time effect @3 mo.
					9.35	8.24	−2.15	NA	≤.007	Patients with a baseline HbA1c ≥ 7 %
										Time effect @3 mo.
							NA	0.22	NS	Patients with a baseline HbA1c ≥ 7 %
										Time effect @3 mo.
36	HS Kim and Jeong 2007 [41] “A nurse short message service by cellular phone…”			✓	8.09	7.59	−1.05^a^	0.11^a^	.008	Group × Time interaction @6 mo.
37	HS Kim and Song 2008 [43] “Technological intervention for obese patients with type 2 diabetes”			✓	8.16	7.66	−1.09^a^	0^a^	.043	Group × Time interaction @6 mo.
							−1.09^a^	NA	≤.050	Time effect @6 mo.
							NA	0^a^	NS	Time effect @6 mo.
38	SI Kim and HS Kim 2008 [73] “Effectiveness of mobile and internet intervention…”			✓	8.16	7.66	−1.49^a^	0.53^a^	.017	Group × Time interaction @12 mo.
							−1.49^a^	NA	≤.050	Time effect @12 mo.
							NA	0.53^a^	NS	Time effect @12mo.
39	Kwon et al. 2004 [74]			✓	7.5	NA	−0.5	NA	≤.003	Time effect @3 mo.
40	Kwon et al. 2004 [45]			✓	7.59	7.19	−0.54	0.33	<0.05	Group effect @3 mo.
							−0.54	NA	≤.050	Time effect @3 mo.
							NA	0.33	NS	Time effect @3 mo.
44	Lorig et al. 2010 [76] (treatment, no reinforcement)			✓	6.5	6.40	−0.03	0.13	0.04	Group effect @6 mo.
	Lorig et al. 2010 [76] (treatment and reinforcement)			✓	6.43		0.02	0.13	0.16	Group effect @6 mo.
	Lorig et al. 2010 [76] (treatment combined)			✓	6.47		−0.01	0.13	0.04	Group effect @6 mo.
46	McCarrier et al. 2006 [77]			✓	7.99	8.05	−0.37	0.11	0.16	Group effect @12 mo.
49	McMahon et al. 2005 [78]			✓	10.0	9.9	−1.6	−1.2	≤.050	Group × Time interaction @12 mo.
50	McMahon et al. 2012 [47] (online care)			✓	9.6	NA	−1.3	NA	<.0001	Time effect @1 year
									NS	Group effect between online care and usual care with web-training @1 year
	McMahon et al. 2012 [47] (telephone care)			✓	9.9	NA	−1.5	NA	<.0001	Time effect @1 year
									NS	Group effect between telephone care and usual care with web-training @1 year
	McMahon et al. 2012 [47] (usual care with web-training)			✓	10.1	NA	−1.7	NA	<.0001	Time effect @1 year
52	Meigs et al. 2003 [49]			✓	8.4	8.1	−0.23	0.14	0.09	Group × Time interaction @12 mo.
55	Moattari et al. 2013 [80]			✓	9.1	9.4	−2.0	−0.6	<.001	Between group @3 mo.
56	Moorman et al. 2012 [81]			✓	8.9	8.9	NA	NA	NS	Between prospective (intervention) vs. retrospective (control) group
57	Musacchio et al. 2011 [82]			✓	6.6	NA	0.2	NA	NP	Patients with a baseline HbA1c < 7.5 % @12 mo.
					7.7	NA	−0.4	NA	NP	Patients with a baseline HbA1c between 7.5 % and 8 % @12 mo.
					8.3	NA	−0.9	NA	NP	Patients with a baseline HbA1c between 8 % and 9 % @12 mo.
					10.0	NA	−2.2	NA	NP	Patients with a baseline HbA1c > 9 % @12 mo.
58	Nes et al. 2012 [83]			✓	7.4	NA	−0.4	NA	NP	@3 mo.
61	Ralston et al. 2009 [38]			✓	8.2	7.9	−0.9	0.2	0.01	Group × Time interaction @12 mo.
62	Ryan et al. 2013 [85]			✓	7.5	NA	−0.6	NA	0.04	Time effect @ 13 mo.
63	Sacco et al. 2009 [48]			✓	8.4	8.5	−1.0	−0.7	NS	Group effect @6 mo.
67	KE Smith et al. 2004 [86]			✓	10.95	NA	−2.22	NA	0.001	Time effect @6 mo.
68	Song et al. 2009 [87]			✓	7.6	7.7	−0.8^a^	−0.4^a^	0.26	Group × Time interaction @3 mo.
69	Stone et al. 2012 [88] (Active care management to care coordination with home telemonitoring)			✓	7.77	NA	0.26	NA	NS	Time effect @ 6 mo.
	Stone et al. 2012 [88] (Active care management to care coordination)			✓	7.97	NA	0.19	NA	NS	Time effect @ 6 mo.
	Stone et al. 2012 [88] (Care coordination to care coordination)			✓	8.56	NA	0.15	NA	NS	Time effect @ 6 mo.
	Stone et al. 2012 [88] (Care coordination to usual care)			✓	8.53	NA	0.31	NA	NS	Time effect @ 6 mo.
71	Tang et al. 2013 [89]			✓	9.2	9.3	−1.1	−1.0	0.13	Between group @1 year
73	Tildesley et al. 2010 [90]			✓	8.8	8.5	−1.2^a^	−0.1^a^	≤.050	Group effect @6 mo.
							−1.2^a^	NA	≤.001	Time effect @6 mo.
							NA	−0.1^a^	0.51	Time effect @6 mo.
76	Yoo et al. 2009 [91]			✓	7.6	7.4	−0.5	0.2	≤.001	Group × Time interaction @3 mo.
77	Yoon and HS Kim 2008 [92]			✓	8.09	7.59	−1.32^a^	0.81^a^	≤.001	Group × Time interaction @12 mo.
65	Seto et al. 2012 [16]	✓	✓		7.3	NA	−0.3	NA	<.001	Time effect @ 8 mo.
4	Bailie et al. 2004 [62]	✓		✓	9.0	NA	−0.2^a^	NA	0.23	Time effect @3 years
11	Cho et al. 2006 [57]	✓		✓	7.7	7.5	−1.0^a^	−0.1^a^	≤.050	Group effect @30 mo.
16	de Grauw et al. 2002 [19]	✓		✓	8.2	NA	−1.1	NA	≤.001	Unpaired *t*-test @6 year
30	Jones and Curry 2006 [50]	✓		✓	7.25	7.13	0.06	−0.18	0.24	Group effect within 16 months
45	MacLean et al. 2009 [20]	✓		✓	7.11	7.03	0.05	−0.02	0.08	Group × Time interaction @32 months
64	Sadur et al. 1999 [22]	✓		✓	9.7	9.6	−1.3	−0.22	≤.0001	Group effect @6 mo. or beyond
28	Holbrook et al. 2009 [28]		✓	✓	7.0	7.1	−0.2	0.2	0.03	Group effect @6 mo.
29	Hurwitz et al. 1993 [72]		✓	✓	10.4	10.3	−0.4	0.3	NP	Group effect @2 year
48	McDiarmid et al. 2001 [51]		✓	✓	8.0	NA	−0.1	NA	NP	Time effect @1 year
53	Meulepas et al. 2007 [30]		✓	✓	7.2	7.4	0	0.6	≤ .050	Group effect @2 year after intervention (baseline 1 year before intervention)
54	Meulepas et al. 2008 [31]		✓	✓	7.3	7.2	−0.2	0.1	<0.05	Group × Time interaction @3 years
72	Thomas et al. 2007 [26]		✓	✓	7.3	7.4	−0.02	−0.01	0.83	Group × Time interaction @ 1 year
75	Yeh et al. 2006 [33]		✓	✓	9.03	8.95	−1.65	−0.92	0.01	Group effect @8 mo.
42	Lin et al. 2007 [29]	✓	✓	✓	7.8	7.7	−0.6	NA	≤.050	Time effect @3 year
							NA	−0.3	0.24	Time effect @3 year

*NS* Non-significant (p-value>0.05), *S* Significant (p-value≤0.05), *NA* Not applicable, *NP* Not provided

Results are differences in mean before and after implementation of intervention except those indicated with the following superscripts

^a^Multiple measurements are presented over time after the intervention in the paper, but the last measurement is used to calculate the difference in this table

The behavioral outcomes, summarized in Appendix 3, include measures related to self-management (SMBG testing, physical activity, foot care, diet, nutrition, self-efficacy, quality of life, and patient satisfaction), attendance to outpatient visits for laboratory tests, vaccinations, primary care and specialty care, adherence to ADA guidelines (annual foot exam, annual eye exam, and processes of care), and acute care utilization (emergency visits, and hospital admissions). Since different measures or tools are used in different studies, we did not provide the numerical values for the changes in outcomes. For example, patient satisfaction is measured using different survey tools. The attendance to laboratory visits are measured using the number of laboratory tests within the next 6 months or 12 months after the intervention, or the percentage of patients who had the recommended laboratory tests within a year. For adherence to recommended laboratory tests, we included the tests considered in that study, and for vaccinations we presented the vaccinations.

In Tables 3, 4, and 5, we summarize the primary outcomes from the studies in Table 2, Appendix 2, and Appendix 3, based on if the interventions were directed at getting patients on the schedule, to the visit, or with the necessary patient information, respectively. Reference numbers of studies with significant outcome findings are bolded. In the following sections, we describe the most notable findings from these studies.

**Table 3:** Summary of outcomes and statistically significant results relating to getting patients on the schedule

Type of intervention	Primary outcomes	Studies analyzing primary outcomes	Studies with significant results	References
Phone Reminder	↓HbA1c	3	3	[16, 19, 29]*
	↓SBP	2	0	[19, 29]
	↓Cholesterol	2	2	[19, 29]*
	↑# HbA1c tests	4	4	[16–19]*
	↑# of provider visits	2	2	[18, 19]*
	↑Eye exam	2	2	[15, 17]*
Letter/Mail Reminder	↓HbA1c	3	1	[20, 50] [12]*
	↓SBP	2	1	[50] [12]*
	↓Cholesterol	3	0	[12, 20, 50]
	↑# HbA1c tests	3	1	[13, 20] [14]*
	↑# of provider visits	2	1	[14] [20]↓*
	↓ED visit rate	1	1	[20]*
	↓Hospitalization rate	1	1	[20]*
	↑Eye exam	3	3	[14, 15, 50]*
Scheduling when necessary while monitoring patient	↓HbA1c	1	1	[22]*
	↑# of provider visits	2	1	[22] [21]*
	↓ED visit rate	1	1	[21]*
	↓Hospitalization rate	2	2	[21, 22]*
	↑Eye exam	1	0	[21]
Open access scheduling	↓HbA1c	1	1	[23]*
	↓Cholesterol	1	0	[23]
	↑# HbA1c tests	1	0	[23]↓*
	↑# of provider visits	1	0	[23]
	↓ED visit rate	1	0	[23]
	↓Hospitalization rate	1	0	[23]

*indicates significant findings with p-value ≤0.05; ↓=decrease, ↑increase

*NP* p-value is not given

**Table 4:** Summary of outcomes and statistically significant results relating to getting patients to the visit

Type of intervention	Primary outcomes	Studies analyzing primary outcomes	Studies with significant results	References
Phone Reminder	↓HbA1c	5	5	[16, 28–31]*
	↓SBP	4	2	[29, 31] [28, 30]*
	↓Cholesterol	4	1	[28, 30, 31] [29]*
	↑# HbA1c tests	5	4	[32] [16, 25, 28, 30]*
	↑# of provider visits	2	2	[27, 28]*
	↓Hospitalization rate	2	1	[27] [32]*
	↑Eye exam	2	2	[30, 32]*
	↑Foot exam	3	3	[28, 30, 32]*
Letter Reminder	↓HbA1c	2	0	[26] [72]^NP^
	↓SBP	1	0	[26]
	↑# HbA1c tests	3	3	[24–26]*
	↑# of provider visits	1	1	[27]*
	↓Hospitalization rate	1	0	[27]
SMS Reminder	↓HbA1c	1	1	[33]*
	↓Cholesterol	1	1	[33]*
Financial incentive	↓SBP	1	1	[34]*
	↑# HbA1c tests	1	1	[24]*
	↑# of provider visits	1	0	[34]↓*
	↓ED visit rate	1	1	[34]*
	↓Hospitalization rate	1	0	[34]

*indicates significant findings with p-value≤0.05; ↓=decrease, ↑increase

*NP* p-value is not given

**Table 5:** Summary of outcomes and statistically significant results relating to collecting patient information

Type of intervention	Primary outcomes	Studies analyzing primary outcomes	Studies with significant results	References
Web-based management with feedback	↓HbA1c	33	26	[68, 70, 77, 87, 89] [36–43, 45, 47, 57, 61, 65–67, 71, 73, 74, 76, 78, 80, 85, 86, 90–92]* [72, 83]^NP^
	↓SBP	10	3	[37, 38, 47, 85, 86, 89] [36, 78, 91]* [71]^NP^
	↓Cholesterol	20	8	[38, 44, 45, 47, 57, 66, 74, 78, 85, 86, 92] [36, 43, 67, 70, 80, 89–91]* [71]^NP^
	↑# of provider visits	3	1	[76, 89] [61]*
	↑QOL	2	0	[85] [83]^NP^
	↑Self-efficacy	2	1	[76] [77]*
Phone/SMS/Mail	↓HbA1c	6	1	[48, 63, 81, 88] [47]* [82]^NP^
	↓SBP	1	1	[47]*
	↓Cholesterol	2	1	[47] [88]*
	↑# of provider visits	2	1	[21] [46]↓*
	↑Eye exam	1	1	[58]*
	↑Foot exam	1	1	[58]*
	↓ED visit rate	2	1	[46] [21]*
	↓Hospitalization rate	2	1	[46] [21]*
	↑QOL	4	2	[58, 84] [46, 63]*
	↑Self-efficacy	2	2	[48**,** 84]*
	↑SMBG testing	4	2	[63, 81] [58**,** 69]*
Decision support; Evidence based guidelines	↓HbA1c	7	2	[29, 49, 50, 62] [28**,** 33]* [51]^NP^
	↓SBP	5	2	[29, 50, 62] [28, 49]*
	↓Cholesterol	5	2	[28, 49, 50] [29, 33]*
	↑# HbA1c tests	5	5	[17, 28, 49, 51, 62]*
	↑# of provider visits	1	1	[28]*
	↑Eye exam	5	4	[49] [17, 50, 51, 62]*
	↑Foot exam	6	6	[28, 49**–**51, 62, 75]*
Registry	↓HbA1c	3	1	[20, 26] [19]*
	↓SBP	2	0	[19, 26]
	↓Cholesterol	3	1	[20, 26] [19]*
	↑# HbA1c tests	6	3	[13, 20, 52] [19, 26, 53]*
	↑# of provider visits	2	1	[19] [20]↓*
	↓ED visit rate	1	1	[20]*
	↓Hospitalization rate	1	1	[20]*
	↑Eye exam	1	1	[52]*
	↑Foot exam	1	1	[52]*
	↑QOL	1	0	[20]
Personal health records	↓HbA1c	2	1	[55] [54]*
	↓SBP	1	0	[54]
	↓Cholesterol	2	0	[54, 55]
	↑# HbA1c tests	1	0	[54]
	↑Eye exam	1	0	[54]
	↑Foot exam	1	1	[54]*

*indicates significant findings with p-value≤0.05; ↓=decrease, ↑increase

*NP* p-value is not given

### On the schedule

For the purpose of this literature review, an intervention that enables a patient to schedule a provider appointment or laboratory test meets criteria for ‘on the schedule’. Review of the literature found limited research studying scheduling interventions as compared to diabetes intervention research pertaining to communication of patient information to the provider. The scheduling interventions, summarized in Table 3, included sending reminders to schedule a provider appointment or laboratory test, scheduling when necessary while monitoring patient information, and open access scheduling to provide same-day access. Although phone reminders were found to be effective for the most part to increase patient attended appointments, impact on clinic outcomes, as with other interventions in this focus group, were mixed and only a few studies discussed proactive appointment scheduling or management.

Grassroots interventions such as letter and phone reminders have been used to remind diabetic patients to schedule a provider appointment or a laboratory test. While the letter reminder, which asked the patients to call and make an appointment, improve the clinical outcomes including HbA1c, and SBP significantly in one study [12], it was not very effective in improving the clinical outcomes in other studies [13]. In a RCT, a letter from the provider was mailed to patients prior to their birthday with a self-care handbook, preventive care checklist and recommendations for routine monitoring and screening resulting in a significantly increased percentage of patients with an HbA1c test, percentage of patients with one diabetes-related provider visit, and percentage of patients with an eye exam within 6 or 12 months after the intervention [14]. In another RCT, patients receiving a phone reminder to schedule an appointment 10 days following a letter reminder had significantly higher return rates for an annual follow-up eye exam than those patients who received only a reminder letter [15]. In a pretest/posttest study, phone calls made by medical assistants to schedule follow-up appointments with the primary care provider significantly improved glycemic control (reduced HbA1c levels) for the patients who returned for their follow-up visit [16]. In another study using RCT, phone calls to schedule an appointment with a pharmacist approximately one week prior to the physician appointment significantly improved compliance to ADA standards of care including percentage of patients who had A1c test, fasting lipid profile, foot exam and vaccinations [17]. An automated outreach call to non-adherent patients advising them to schedule an appointment significantly improved the percentage of patients with a provider visit and with HbA1c test for those patients who were successfully reached [18]. In a multi-center cross-sectional study, a phone call to reschedule after a no-showed appointment for a periodic provider visit resulted in significantly increased patient attendance to annual provider review, and those patients who attended their annual review had significantly lower fasting blood glucose [19].

Different than the studies that consider reminders to patients only, one study combined reminders to the patient with reminders to the provider [20]. In a RCT, faxed reminders were sent to the provider for patient overdue laboratory tests and letter reminders were sent to the patients with a warning of overdue laboratory tests. Even though the decrease in HbA1c and LDL of the intervention group when compared to control group was not significant, the number of emergency visits and number of hospital days per year were reduced significantly [20].

Comprehensive diabetes management programs that are used to monitor patient status can also be used to facilitate scheduling of patients for their provider visits. In a retrospective cohort study, the care coordinator regularly reviewed patient uploaded information such as SMBGs and scheduled provider appointments when appropriate, resulting in significantly decreased percentage of patients with at least one emergency visit and hospital admission [21]. In another RCT, a nurse reviewed self-management by phone at regular intervals, and a multidisciplinary care team provided both group visits every month for 6 months and individual visits after patient self-referral or referral by another care team member. The HbA1c levels and number of hospital admissions significantly reduced for the intervention group [22].

Open access, a scheduling strategy that offers same-day appointments for patients, can aide patients in scheduling a provider appointment and needed laboratory testing [23]. A drawback with this type of scheduling strategy is that the patient has the responsibility to initiate the next appointment at the appropriate time as specified in diabetes practice guidelines. If the patient forgets the timing of laboratory tests and provider visits, and the clinic does not send reminders to the patient for scheduling their appointments, open access scheduling might reduce compliance to diabetes management guidelines. One retrospective cohort study showed that open access scheduling was associated with significant decrease in HbA1c and urine microalbumin testing [23]. Even though HbA1c levels, and the number ED visits and hospitalizations did not change significantly with open access scheduling, the study suggested that scheduling process should be adjusted for patients with diabetes to improve diabetes processes of care (HbA1c, LDL, urine microalbumin testing) [23].

### To the visit

Attendance to provider appointments and laboratory testing is a necessary component for implementation of diabetes preventive care. Interventions facilitating patient attendance to the scheduled provider appointments or laboratory testing meet criteria for the focus area ‘to the visit’. Review of the literature found fewer studies discussing interventions to facilitate getting the patients to their provider visits as compared to diabetes intervention research pertaining to communication of patient information to the provider. The interventions that are used to improve attendance to the scheduled visits include letter, phone call, and SMS reminders, and financial incentives, as summarized in Table 4. Phone and mail reminders were the interventions most studied to facilitate patient appointment attendance with positive clinical outcomes. More studies are needed to determine if SMS and web-based appointment reminders and financial incentives can also improve provider visit attendance.

Our literature review showed that letter reminders to patients regarding lab appointment information were associated with significantly increased average number of HbA1c tests within the study period, number of patients who had HbA1c test within 6 months, and percentage of patients who completed the HbA1c test within a certain period after the reminder [24–26]. In a RCT, letters recommending appropriate laboratory testing were automatically mailed quarterly to patients without HbA1c tests in the last six months or without LDL within the last twelve months resulting in significantly increased number of patients who had HbA1c test within 6 months and LDL test within 12 months [26]. Letter reminders one week before the scheduled provider appointment significantly increased the number of provider visits and reduced the number of hospitalizations in another RCT [27].

Phone reminders to patients regarding provider visits and laboratory testing resulted in improved HbA1c levels [16, 28–31]. One study showed that monthly phone reminders to patients in the intervention group regarding laboratory or provider scheduled appointments resulted in significantly decreased HbA1c levels and systolic blood pressure in the intervention group when compared to the control group [28]. Two studies where the medical assistant or the secretary called each patient before their scheduled appointment day to remind them of the appointment were associated with significantly decreased HbA1c [16, 29] and LDL levels [29]. In two studies using a controlled, non-randomized before/after design, a Diabetes Support Service (DSS) called patients in the intervention group to remind them of scheduled appointments for laboratory testing, foot exam, fundus photography and scheduled appointments with the dietician and diabetes nurse. The intervention was associated with a significantly increased percentage of patients with at least four HbA1c tests a year [30] and significantly lower HbA1c levels in the intervention group when compared to the control group [30, 31].

Letter reminders combined with phone reminders of the date and time of the patient’s provider appointment or laboratory test resulted in improved health outcomes [25, 27, 32]. One study showed that a recall card system and phone call reminding patients of their scheduled follow-up appointment resulted in significantly increased the percentage of patients who had HbA1c within the last 6 months and LDL tests within the last 12 months, significantly decreased the percentage of patients hospitalized in the last 12 months, and significantly increased the percentage of patients with foot exams and eye exams in the last 12 months [32].

Web-based programs associated with self-management can successfully remind patients regarding provider appointments or laboratory testing. A RCT used a web-based system to improve self-management education, and used emails combined with short message service (SMS) to send reminders one week before the follow-up visit, and to remind the time of the HbA1c test if it is more than three months overdue [33]. This web-based education management system combined with email and SMS reminders resulted in significantly decreased HbA1c and total cholesterol levels in the intervention group compared to control group [33].

Financial incentives used with other interventions have the potential to improve attendance to scheduled visits or needed lab tests. In a quasi-experimental study, a reminder letter was sent to patients for the completion of lab tests, and were offered and provided a gas card when the tests were completed [24]. The study showed that the reminder letter combined with a financial incentive increased the number of HbA1c tests significantly [24]. In another study, structured group visits facilitated by a diabetes educator were used as the main intervention [34]. A $10-incentive was provided to the patients for each group visit they attended [34]. Group visits combined with financial incentive achieved an overall attendance of 78.4 % to group visits, and significantly reduced SBP levels and number of ED visits per year [34].

### Patient information

ADA, Healthy People 2020 and the Chronic Care Model recognize the primary importance and responsibility of the patient in self-managing their diabetes care and collaborating with their providers to set treatment and goals for improved health outcomes [4, 5, 35]. Interventions that aide the patient in communicating important information regarding SMBGs, daily diet and nutrition, exercise or physical activity, medication information and compliance, and patients’ needs to their provider or health care team meet conditions for the focus area ‘with patient information’ (see summary of interventions and findings in Table 5). This focus area of the literature review provided the greatest number of research studies when compared to the other two focus areas, ‘on the schedule’ or ‘to the visit’. Systems with routine monitoring of patient information, managing patient medications and supporting patients’ goals whether web-based, SMS, or Electronic Health Record (EHR) with interfaced registry, consistently showed improved patient clinical outcomes.

This literature review identified multiple studies using web-based diabetes management interventions with care manager feedback. In a RCT study, patients entered SMBG readings, exercise amounts, weight changes, blood pressure, and medication data via a web portal [36]. The study nurse monitored self-management changes, and contacted patients using email or chat to make recommendations [36]. The intervention resulted in significantly decreased HbA1c, systolic blood pressure and total cholesterol levels in the intervention group as compared to the control group who visited their provider for usual care [36]. In another RCT study, a nurse contacted patients biweekly for a 30 min video conference to review biometric data uploaded to the web-based self-management module and discuss patients’ problems in managing the disease [37]. The intervention significantly decreased HbA1c levels in the intervention group [37]. Another study, which used randomized, single-centered, controlled trial with parallel group design, evaluated a web-based program used by patients to review their online medical records, upload their SMBG levels, enter information about their exercise, diet and medication, and send secure emails to the care manager [38]. The care manager reviewed SMBG readings, guided health behavior, adjusted medications, and responded to patients’ messages [38]. This web-based program, which provided ongoing tracking and documentation of patients’ needs and care, decreased HbA1c levels significantly [38]. Seven studies combined web-based diabetes management program with SMS and were associated with significantly decreased HbA1c levels for the intervention group after implementation [39–45]. In six of those studies using quasi-experimental pretest/posttest method conducted by the same research group, the nurse researcher reviewed uploaded patient data on the website, integrated patient clinical information into the patients’ EHRs, provided education for self-management and sent weekly medication adjustment advice to the patient via SMS and internet [39–44].

Two studies showed that patients using a telephone data line to answer care coordinator’s questions regarding daily SMBG readings, medication compliance and symptoms which were forwarded to the provider were associated with significantly increased quality of life (QOL) [46] and significantly decreased the percentage of patients with emergency visits and hospital admissions [21]. One study showed that patients receiving bi-weekly phone calls to review glucose and blood pressure readings had significantly reduced HbA1c and SBP levels [47]. Another study showed weekly phone coaching for goal setting and self-management significantly improved self-efficacy, diet, exercise, and foot care [48].

This literature review showed that the tools enabling decision support at the time of patient contact could improve compliance with preventive care services. A disease management application, which displayed trended electronic laboratory data linked to evidence-based treatment recommendations, resulted in significantly increased average number of HbA1c and LDL tests per year in a RCT study [49]. Patient data entered into Personal Digital Assistant (PDA), which enabled the tracking of evidence-based guidelines and provided reminders of due or overdue tests to providers at each patient visit, improved compliance to eye and foot exams [50]. The Diabetes Questionnaire and Reminder sheet, which is completed by the patient at check-in and reminded providers to check feet and update diabetes care flow chart used to document dates of preventive services in patient’s chart, increased the number of HbAc1 tests, and compliance to eye and foot exams [51].

The utilization of an EHR driven diabetes registry within an integrated delivery system can improve diabetes health outcomes. A multicenter cross-sectional study showed that a computerized registration with templates for recording patient data from quarterly or annual diabetes visits integrated with patient’s EHR resulted in significantly increased percentage of patients with HbA1c tests, and significantly decreased HbA1c, total cholesterol and triglycerides levels [19]. In a RCT study, a laboratory-based registry was used to fax and/or mail laboratory results, reminders of overdue laboratory tests, and quarterly population reports to providers, and to mail reminders for overdue tests and alerts for elevated test results to patients [20]. The integration of registry with patient and provider decision support decreased acute care utilization significantly, but did not decrease HbA1c level significantly [20]. A diabetes registry can be used to generate provider performance audits or provider patient panel reports to provide feedback regarding achievement of diabetes care guidelines including HbAc < 7.0 %. In three studies, these reports were shown to be associated with significantly improved diabetes processes of care (percentage of patients who had HbA1c test in the last six months, annual LDL cholesterol test, annual dilated eye exam, annual foot exam, and annual influenza vaccine) [26, 52, 53].

Personal patient held records summarizing goals, medical and laboratory outcomes for the year can assist both patients and providers as they organize individualized diabetes treatment plans. One study using clustered RCT showed that the intervention group utilizing patient-held health records resulted in significantly decreased HbA1c levels in the intervention group as compared to the control group [54]. However, web-based personal health records that allowed patients to review their medication lists, most recent test results and current treatments before the visit did not improve HbA1c levels in another RCT study [55].

## Discussion

**Fig. 2:**
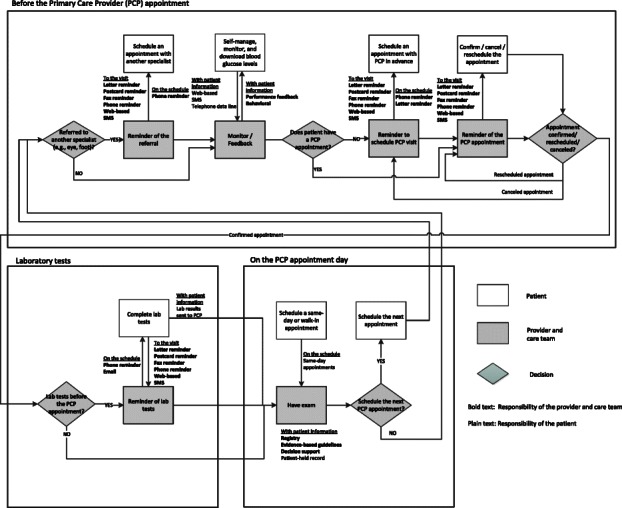
Diabetes outpatient care delivery process

ADA and Healthy People 2020 recommended diabetic patients have routine laboratory tests and provider visits at regular intervals [4, 5]. This literature review evaluated diabetes interventions, their effectiveness and resultant health outcomes, focusing upon the areas of scheduling the patient, getting the patient to their provider visit, and having patient information available to the provider. Figure 2 summarizes our findings by illustrating patient flow through the complex medical outpatient care delivery process with all potential interventions identified in this review. More specifically, Fig. 2 shows various components of diabetes outpatient care delivery, identifies phases of the process when interventions could be applied, identifies potential types of multifaceted interventions that could be utilized, and distinguishes whose responsibility it is for successful navigation through each phase of the care delivery system, e.g., provider and health care team versus patient.

### Identifying gaps and highlighting future research opportunities

Diabetes management requires continuous monitoring and routine provider visits and laboratory tests [4]. This literature review showed that routine visits are either scheduled in advance or reminders are sent to patients to schedule their next appointment. When appointments are scheduled in advance, the attendance to scheduled visits might decrease as the lead time between the time the appointment is scheduled and the actual appointment time increases [56]. Therefore, advanced scheduling should be integrated with other interventions used to improve attendance to scheduled visits. In addition, clinics are moving from advanced scheduling to open access scheduling to reduce waiting times and improve access to care. However, one study showed that open access scheduling negatively affected the process outcomes for diabetes patients [23]. The mixed findings demonstrate the importance of provider or care team initiated interventions such as reminders sent to patients to schedule an appointment, or monitoring of patient information and scheduling when needed. The literature review showed that implementing automated or personalized phone reminders, which are relatively simple interventions and easy to employ by provider practices, are very useful in improving appointment making and attendance behavior.

Web-based diabetes management tools are used to continuously monitor patient information and provide feedback to the patient. The continuously monitored patient information might include SMBG readings, patient medication use, blood pressure, weight, and nutrition or daily calorie intake. The degree of interaction with patients might range from providing feedback about SMBG readings by care manager to online coaching and structured counseling by diabetes specialist or nurse practitioner. The web-based systems can also be used to integrate laboratory testing and clinical information into patient’s EHR. Web-based tools require patient, provider and care team involvement. Although a few of the studies discussed ease of use of the web-based interventions by patients and review patient satisfaction [46, 57, 58], none of the studies in this literature review discussed the ease of use for providers, provider satisfaction, or impact to the provider workload. Most of the web-based interventions using care manager monitoring and feedback used small sample sizes and did not discuss the direct and indirect costs and ease of implementation of the interventions for larger populations. More studies discussing provider workload and information regarding costs of the intervention may aid a practice in determining which inventions are most suited for their practice.

Selective financial incentives can improve quality of health services [59]. Three of the studies in this literature review incorporated financial incentives within the intervention. In one study, a gas card was given to patients after the completion of laboratory tests (HbA1c and LDL) and was associated with significantly increased laboratory testing when combined with a written reminder [24]. Another study discussed monetary incentives to providers for improving diabetes processes of care as demonstrated by significant increases in the percentage of patients with ideal glucose levels (HbA1c < 7.0 %) when combined with provider feedback and computerized reminders [53]. More research is necessary to determine the effect of both patient and provider financial incentives on patient health outcomes.

Tailoring the interventions according to patient population characteristics, needs, capabilities, and skills is an important factor that should be considered while choosing the set of interventions for implementation. For example, a web-based self-management program may not be as appropriate for elderly patients who may not be as comfortable with computer usage as a younger patient. The patients who have cellphones may not answer phone calls, but respond to SMSs. With the increasing use of smartphones, the patients might have regular access to email. However, underserved populations may not even have a regular phone or minutes to answer phone calls/SMSs in their cellphones. Changing technology and patient preferences with regard to contact/communication should be considered when determining the future interventions to improve usage and effectiveness. Research evaluating the usage of interventions tailored for different patient groups is needed. Web-based tools with continuous monitoring can be used to categorize patients according to risk groups. Structured counseling and proactive scheduling of provider appointments might be used for high-risk patients to reduce the acute care utilization.

This literature review identified several interventions that improve appointment management and preparation. While impact of interventions on several clinical and behavioral outcomes is evaluated in these studies, effectiveness of interventions is not evaluated from a systems perspective. In other words, the interventions in the literature we reviewed appeared to be examined in isolation when they may, in fact, have repercussions throughout a provider’s practice and patient population. Other factors such as ease of use by patients and providers, applicability of the intervention for larger populations and across other chronic diseases, and the cost of implementation are important concerns that may influence providers’ decisions about adopting interventions in their practices. Research is needed that includes a more systematic view of the interventions and their implications beyond patient outcomes.

The methodologies used in the reviewed papers vary widely (including RCTs, quasi-experimental, pretest-posttest, retrospective cohort, non-randomized controlled trial, nested randomized trial, etc.). Even though RCT is considered as the best method in terms of strength and validity of the results, the reviewed studies that use other methods usually consider an intervention that can easily be implemented in large patient populations. These interventions include phone, letter/mail and SMS reminders to schedule an appointment or remind a scheduled appointment, and diabetes registries, and decision support systems to improve compliance to diabetes management guidelines. Since these interventions use large sample sizes, the included studies prove the applicability and impact of these interventions. For the studies that consider using a web-based system with care coordinator feedback, RCTs are used with smaller sample sizes. Even though RCTs show the positive impact of such kind of an intervention, the small sample size might be an indicator of the difficulty of implementation due to the cost of the intervention.

### Limitations

While the search in this literature review was conducted using several key databases and references were cross-checked, there may be publications not incorporated in the review because of the MESH terms used and inclusion criteria utilized. Only studies published in English were included which may create a chance for potential bias. All studies included in the literature review were peer-reviewed publications. Although some interventions may be dated due to inclusion of studies published as early as 1987, less sophisticated interventions may have the same or better payoff and achieve similar goals at less cost and complexity in implementation.

One limitation of this literature review is that a meta-analysis was not performed due to inconsistency of the reported outcomes [60]. The included studies report a wide range of outcomes. Especially, the behavioral outcomes in Appendix 3, are not consistent. The measures related to self-management use different survey tools to assess patient satisfaction, quality of life, self-efficacy, etc. Other measures such as lab tests completed, vaccinations, provider visits, hospitalizations, and ED visits, are reported as either percentages or numbers (i.e. “percentage of patients who had ED visits” vs. “number of ED visits”). For clinical outcomes, the studies might report time effect, group effect, or time × group effect, which is again not consistent from study to study. Some studies did not use a control group or did not provide enough information before or after the intervention. This inconsistent reporting of wide variety of outcomes, and limited number of studies representing each outcome made the meta-analysis impractical.

## Conclusions

The literature review showed that interventions from the simplest phone and letter reminder for scheduling or prompting of the date and time of an appointment to more complex web-based multidisciplinary programs with patient self-management can have a positive impact on clinical and behavioral outcomes for diabetes patients. Multifaceted interventions aimed at appointment management and preparation during various phases of the medical outpatient care process may provide a fail-safe against diabetes patients falling through the cracks of a fissured health care delivery system and maximize patient-provider limited time while obtaining the best possible disease management. While the overall results from this review suggest that interventions associated with appointment management and preparation result in better patient outcomes, an overwhelming absence of financial information in the reviewed studies may inhibit implementation. Indeed, practices may see an increase in costs associated with dedicated care managers and information technology support. Patients, and their insurers, may see an overall decrease in the costs of care when proper disease management is practiced. Unfortunately, these cost offsets may not be within the same cost center, and therefore, the providers paying for the interventions may not realize the cost benefits of enhanced patient well being. Future research must address these cost concerns and new policies may be necessary to ensure that interventions are beneficial for patients and providers.

This literature review also revealed that the trend of diabetes care is moving toward frequent monitoring of patient data and fluid management of patient diabetes care. Complex web-based systems are being overseen by an intermediate care manager, which may be an advance practice nurse, physician assistant or diabetes educator for 1) monitoring of SMBG levels, laboratory tests, medication compliance, diet and nutrition, physical activity and, 2) directing changes in patient care based on patient information. This intermediate care manager also directs the flow of patient information to provider, specialist and other members of the multidisciplinary health care team. The questions are whether the future of diabetes care and this type of continual monitoring will concentrate provider visits more toward those patients whose diabetes are not well-controlled or have a higher severity and what impact this change will have on overall diabetes outcomes. It seems reasonable that with the predicted increases in diabetes incidence and the already overloaded provider schedules that new strategies are needed to ensure access to care for all diabetes patients. Such strategies must include technical innovation that moves beyond the clinic visit, including continuous monitoring and risk assessment using emerging sensor technologies and smart algorithms, (semi) automated selection, execution, and tracking of interventions, learning algorithms to customize patient care plans, and gamification strategies to motivate and engage patient behaviors. Further, comprehensive cost-benefit analysis must become more widely accepted and practiced. The short and long term costs of interventions (capital, operational, maintenance, cyberinfrastructure, etc.) must be balanced against expected benefits from all stakeholder perspectives including patient access, outcomes, and satisfaction, clinic performance and provider utilization, inpatient usage patterns, reimbursement policies, and overall sustainability of the healthcare system. These strategies must be part of the larger, on-going efforts to transform healthcare delivery from being an uncoordinated assortment of specialties and special interests, supported by fee for service, to an integrated and holistic system that provides value to patients through prevention, early diagnosis, avoidance of chronic complications, and excellent therapy.

## Appendix

**Table 6:** Detailed information about interventions

Author		Intervention description	Other information
1	Anderson et al. 2003 [15]	On the schedule: Standard (control) and intensive personalized (intervention) groups received reminder letter with date, time, location of eye clinic, toll-free number one month before annual exam to schedule an appointment.	IP follow-up group: phone discussion about diabetic eye disease and transportation arrangement to exam.
		Intensive personalized (IP) group received phone call if appointment not scheduled within 10 days of reminder letter date.	
		To the visit: Not applicable (NA)	
		Patient information: NA	
2	Austin and Wolfe 2011 [24]	On the schedule: NA	NA
		To the visit: Pilot group received reminder letter signed by physician to go to the clinic to have HbA1c or LDL-C tests and are offered a gas card if they receive the tests.	
		Patient information: NA	
3	Avdal et al. 2011 [61]	On the schedule: NA	NA
		To the visit: NA	
		Patient information: Intervention group entered self-measured blood glucose (SMBG) tests, accessed education, and could send messages to researcher through web site. SMBG graphics and profile were available to patient via web site.	
4	Bailie et al. 2004 [62]	On the schedule: Scheduling guideline services integrated with computerized information system identifying patients due for scheduled services.	NA
		To the visit: NA	
		Patient information: Audit of guideline adherence per participant, e.g., target blood pressure 130/80 mm Hg, percentage with HbA1c <7 %. Reminder to providers when patients’ scheduling services due.	
5	Benhamou et al. 2007 [63]	On the schedule: NA	NA
		To the visit: NA	
		Patient information: Patients downloaded SMBG levels to SMS weekly and received medical feedback. Data transmitted to software module creating and managing patient files on database.	
6	Bond et al. 2006 [64]	On the schedule: NA	NA
		To the visit: NA	
		Patient information: Participants accessed website to: access library, receive on-line counseling, receive tailor self-management instruction, participate in weekly problem-solving discussion with nurse, use bulletin board to post goals, and enter SMBG, medication, diet, weight and BP.	
7	Bond et al. 2007 [36]	On the schedule: NA	Intervention group: weekly online education discussion; Control group: access to educational materials via classroom or internet.
		To the visit: NA	
		Patient information: Online (asynchronous [email and bulletin board] and synchronous [instant messaging and chat]) with study nurse. Participant entered SMBGs, exercise, weight, blood pressure, and medication via web portal. Study nurse accessed participants’ logs monitoring self-management patterns. Study nurse contacted participant via email or chat.	
8	Carter et al. 2011 [37]	On the schedule: NA	Intervention group: Access to health education module with culturally age-appropriate education through videos and web sites, and social networking module linking intervention participants.
		To the visit: NA	
		Patient information: Self-management module: Nurse contacted patients biweekly for 30 minutes by video conference and reviewed uploaded data while patient viewed self-management video. Nurse and patient discuss data and behavior-change strategies. Patients would discuss problems in managing disease, e.g., medication side effects, and nurse provided feedback. Nurse transmitted data to patient’s EHR (electronic health record). Provider transmitted updated treatment plans, lab results and other orders via portal to nurse and patient.	
9	Cavan et al. 2003 [65]	On the schedule: NA	NA
		To the visit: NA	
		Patient information: Patients used DiasNet computer model to display and analyze SMBG levels, and problem solve via internet. Data was analyzed and discussed in weekly group sessions.	
10	Cherry et al. 2002 [46]	On the schedule: NA	Patients received free blood glucose monitoring equipment.
		To the visit: NA	
		Patient information: Patients answered daily questions (changes in feet, blood sugar and medication) with Health Buddy (phone tool). Care manager using browser-based tool could automatically risk stratify information, forward patient information to provider, make provider referrals and reinforce self-management.	
11	Cho et al. 2006 [57]	On the schedule: Intervention and control patients scheduled for outpatient visits every 3 mos.	Both groups received diabetes management, nutrition, exercise, and blood glucose self-monitoring education.
		To the visit: NA	
		Patient information: Intervention group uploaded glucose levels, medications, BP and weight to web. Clinical instructors reviewed information daily and sent recommendations every 2 weeks. Medication changes referred to researcher and self-management or lifestyle changes referred to nurse or dietitian.	
12	Cho et al. 2009 [66]	On the schedule: NA	NA
		To the visit: NA	
		Patient information: Participants using diabetes phones transmitted SMBG levels to web server automatically, received provider messages via SMS. Participants using internet entered SMBG levels on individual web charts, used self-management program, communicated with provider. Both groups received visual display graphs of data and encouragement if no SMBG entered > 1 week.	
13	Cho et al. 2011 [67]	On the schedule: NA	NA
		To the visit: NA	
		Patient information: When patients visited the public healthcare post, nurses measured blood glucose level with a PDA for both intervention and control group. For the intervention group, the glucose levels and other health information were uploaded to a remote diabetes center; physicians at diabetes center performed problem assessment and made recommendations for patients. Nurses contacted the patients and educated intervention group patients according to physician instruction.	
14	Chumbler et al. 2005 [21]	On the schedule: Care coordinator facilitated scheduling provider appointment if necessary.	NA
		To the visit: NA	
		Patient information: Intervention group used phone data line to answer questions (symptoms, behavior, and knowledge). Patient data downloaded to care coordinator’s desktop daily and patients contacted via audio-visual conferencing.	
15	Ciemins et al. 2009 [52]	On the schedule: NA	NA
		To the visit: NA	
		Patient information: Diabetes registry integrated with EHR identified diabetes patients prior to office visit, staff able to print patient diabetes care summary sheet for provider, and patient report cards for patients.	
16	de Grauw et al. 2002 [19]	On the schedule: Office assistant contact patients who do not come in for visits at regular intervals.	Feedback at practice and physician level.
		To the visit: NA	
		Patient information: Registry records process and outcome measures from visits into EHR.	
17	Derose et al. 2009 [25]	On the schedule: NA	NA
		To the visit: Automated reminder system for patients with overdue lab tests, included phone calls and/or letters. Interventions are: I.1 Letter, I.2 Letter-Call, I.3 Letter-Call-Letter, I.4 Call, I.5 Call-Letter	
		Patient information: NA	
18	Dijkstra et al. 2005 [54]	On the schedule: NA	NA
		To the visit: NA	
		Patient information: Diabetes passport (PHR) summarizes personal goals, medical or lab outcomes for each year.	
19	Edelman et al. 2010 [34]	On the schedule: Intervention groups with 7 to 8 patients meeting every 2 months.	NA
		To the visit: Received $10 for group visit attended for travel costs.	
		Patient information: Structured group interactions facilitated by diabetes educator with pharmacist and physician adjusting medications based on HbA1c and BP.	
20	Edwards et al. 2012 [17]	On the schedule: Patients are contacted by telephone to schedule an appointment with a pharmacist in Diabetes Assessment Service (DAS) approximately 1 week prior to the physician appointment.	NA
		To the visit: NA	
		Patient information: Pharmacist completed the ADA standards of care including measurement of HbA1c and fasting lipid panel (total cholesterol, LDL, HDL, and triglycerides); a comprehensive monofilament foot exam; administration of pneumococcal and influenza vaccinations; collection of urine sample for screening for microalbumin; referral for funduscopic eye exam; medication history focusing on adherence to prescribed antidiabetic, antihypertensive, and antihyperlipidemic medications and aspirin. The results of tests and any pharmacotherapy recommendations are documented in the patient’s EMR. The note is routed to the physician for review prior to the next appointment.	
21	Farmer et al. 2005 [68]	On the schedule: NA	Both groups given blood glucose monitor
		To the visit: NA	
		Patient information: Both groups given mobile phone; SMBG levels, food intake, insulin dose, and activity levels automatically transmitted to server and made available to patient by web. Intervention group received real time clinical advice and structured counseling from diabetes specialist nurse.	
22	Fischer et al. 2011 [13]	On the schedule: Mailed patient report card reminding patient to schedule appointment if ≥ 2 mos since last provider visit.	NA
		To the visit: NA	
		Patient information: Point-of-care patient report cards generated automatically at visit (and mailed quarterly) included patient performance compared to national targets. Medical assistants encouraged patients’ self-management goals. Quarterly provider performance report card generated from the registry.	
23	Fischer et al. 2012 [69]	On the schedule: NA	The PRM system sends text messages to patients automatically according to an established schedule, and processes responses for appropriate action based on established threshold values.
		To the visit: Patients received text message appointment reminders 7, 3, and 1 days before appointments.	
		Patient information: Patients received blood sugar reading requests every Monday, Wednesday, and Friday. The fasting blood sugar values outside the range of 70 to 400 mg/dL were automatically flagged in PRM and routed to a work queue. A registered nurse reviewed all flagged messages, contacted patients by telephone for follow-up assessment, presented out-of-range values to a physician, and ensured that both telephone encounters and patient-reported blood sugar measurements were appropriately documented in the medical record.	
24	Glasgow et al. 2003 [70]	On the schedule: NA	Peer support
		To the visit: NA	
		Patient information: All groups received information-based diabetes self-management website providing coaching, resources and graphical feedback based on transmitted SMBG levels and diet. Peer information exchange, coping strategies, emotional support, and 5 electronic newsletters.	
25	Glasgow et al. 2004 [58]	On the schedule: NA	NA
		To the visit: NA	
		Patient information: Diabetes Priority Program touchscreen assessment and feedback completed. BP, cholesterol, feet exam, microalbumin, dilated eye exam, dietary, physical activity, and smoking behavior and self-management goals data entered creating patient’s personalized action plan and summary of needed medical procedures printout.	
26	Grant et al. 2008 [55]	On the schedule: NA	Evaluate treatment intensification
		To the visit: NA	
		Patient information: Intervention group used PHRs prior to visit with ability to review and edit medications, self-management goals/limitations, view laboratory results and generate diabetes care plan electronically submitted to physician before next appointment.	
27	Harno et al. 2006 [71]	On the schedule: NA	Home care link free of charge
		To the visit: NA	
		Patient information: Intervention group downloaded SMBG levels into regional database using modem. Self-management system allowed diabetes team to transmit SMS test messages to patients’ mobile phones and internet access.	
28	Holbrook et al. 2009 [28]	On the schedule: NA	NA
		To the visit: Patients received monthly phone reminders for medications and for laboratory and provider visits.	
		Patient information: Most recent laboratory results and other diabetes risk factors (e.g., feet check, smoking and physical activity) available to patient and provider at time of visit. Brief, prioritized messages of advice sent to patient by provider based on automated risk analysis.	
29	Hurwitz et al. 1993 [72]	On the schedule: NA	NA
		To the visit: Database, which sends requests to patients to provide laboratory testing (6 monthly prompt) and optometrist exam (12 monthly prompt).	
		Patient information: Laboratory results incorporated into PHR, sent to patients and request for provider within 10 days (elevated blood glucose 3 days). Lack of feedback (including optometry) prompts phone/letter reminder to provider and letter reminder to patient.	
30	Jones and Curry 2006 [50]	On the schedule: Reminder for recommended care based on practice guidelines and scheduling for services (mailed quarterly).	NA
		To the visit: NA	
		Patient information: Data entered into PDA at each visit: HbA1c, hepatic enzymes, weight, systolic and diastolic BP, and date of glucometer correlation. Clinical practice guideline recommendations tracked: dates/results of last lipid panel, nephropathy screen, eye exam, foot exam, last influenza and pneumococcal vaccinations, last diabetes education, dietician education, and smoking cessation education if needed. Reminder of due or overdue guideline recommendations to provider at each patient visit.	
31	HS Kim et al. 2005 [44]	On the schedule: NA	NA
		To the visit: NA	
		Patient information: Intervention group entered SMBG values and drug information to website. Nurse researcher reviewed entered data and integrated EHR data (smoking habits, body mass index (BMI), blood pressure (BP) and laboratory results), sending recommendations to patient by SMS and internet. Medication changes were communicated to patients’ providers. Education provided and reinforcement of diet, exercise, foot care, medication adjustment and self-management by SMS and internet. If no patient self-monitored blood glucose (SBMG) data entered on website for > 1 week, warning message was sent to patient via internet.	
32	HS Kim et al. 2006 [42]	On the schedule: NA	NA
		To the visit: NA	
		Patient information: See H. Kim et al. 2005 [44] (Index # 31)	
33, 34	HS Kim 2007 [39, 40]	On the schedule: NA	NA
		To the visit: NA	
		Patient information: See H. Kim et al. 2005 [44] (Index # 31)	
35	HS Kim and Jeong 2007 [41]	On the schedule: NA	NA
		To the visit: NA	
		Patient information: See H. Kim et al. 2005 [44] (Index # 31)	
36	HS Kim and Song 2008 [43]	On the schedule: NA	NA
		To the visit: NA	
		Patient information: See H. Kim et al. 2005 [44] (Index # 31)	
37	SI Kim and HS Kim 2008 [73]	On the schedule: NA	NA
		To the visit: NA	
		Patient information: See H. Kim et al. 2005 [44] (Index # 31)	
38	Kirsh et al. 2007 [12]	On the schedule: A letter is sent informing the patient that he/she had suboptimal diabetes measures and inviting the patient to call and make an appointment.	NA
		To the visit: NA	
		Patient information: NA	
39	Kwon et al. 2004 [74]	On the schedule: NA	NA
		To the visit: NA	
		Patient information: Intervention group entered SMBG levels, medication, BP, weight, diet, exercise or hypoglycemic events on web. Providers could review data e.g., past history, family history, smoking, anthropometry, BMI, BP, and lab data. After integration patient data, providers sent recommendations via individual EHR and answered questions. Nurses reviewed lifestyle changes, exercise and dietitians reviewed nutrition via EHR. If no patient SBMG data entered on website for > 1 week, warning message sent via internet.	
40	Kwon et al. 2004 [45]	On the schedule: NA	NA
		To the visit: NA	
		Patient information: Participants entered SMBG levels, medication, and hypoglycemic events on web. Patient questions about medication, diet, and exercise posted through specialized electronic chart on web. SMBG levels also sent using SMS. Providers sent recommendations about medications according to SMBG. Dieticians and nurses provided nutrition and exercise consults on web.	
41	Lafata et al. 2002 [14]	On the schedule: Letter from provider mailed to patient for birthday with felicitations, advise routine appointments, screening and laboratory tests, and a self-care handbook, and preventive care checklist.	NA
		To the visit: NA	
		Patient information: NA	
42	Lin et al. 2007 [29]	On the schedule: Intervention group scheduled for individual 30- minute appointments instead of default 15-minute appointments every 3 mos. A secretary telephoned each patient before scheduled appointment day to arrange for routine blood work one week before the appointment.	NA
		To the visit: A secretary telephoned each patient before scheduled appointment day as reminder of appointment, to bring medications and SMBG log books.	
		Patient information: Standardize diabetic flow sheet according to Canadian Diabetes Association’s guidelines used to record patient information.	
43	Litzelman et al. 1993 [75]	On the schedule: NA	Behavioral contract for desired foot-care
		To the visit: NA	
		Patient information: Nurse-clinicians conducted educational sessions covering foot-care behavior. Intervention group received postcard reminder of desired foot-care behavior. Providers received informational flow sheet providing patient-specific risk factors, foot-care practice guidelines, diagnostic work-up, treatment and referral recommendations.	
44	Lorig et al. 2010 [76]	On the schedule: NA	NA
		To the visit: NA	
		Patient information: Intervention group utilized web diabetes self-management program: ‘The Learning Center’ (educational material), weekly queries for problems and to set action plan, a ‘Discussion Center’ (interactive, threaded), ‘Tools’ (exercise, medication, meal planning and SMBG logs), ‘Post Office’ (private email to facilitator, and ‘Help’ (also available by phone).	
45	Maclean et al. 2009 [20]	On the schedule: The Vermont Diabetes Information System (VDIS), a lab based registry, sent provider faxed reminders and mailed patient reminders for overdue lab tests.	NA
		To the visit: NA	
		Patient information: Provider decision support with faxed lab results flow sheets and mailed quarterly population reports for peer comparisons. Mailed alerts for elevated test results.	
46	McCarrier et al. 2009 [77]	On the schedule: NA	NA
		To the visit: NA	
		Patient information: Intervention group received 1-hour consultation with nurse practitioner and 1:1 web module instruction. Website allowed patient to view their EMR, enter SMBG values, trend daily medication, nutrition, and exercise, create action plan, and use educational resources.	
47	McDermott et al. 2001 [32]	On the schedule: NA	NA
		To the visit: Trained healthcare workers managing a paper-based recall and reminder system for follow-up	
		Patient information: Staff training in checking weight, BP, visual acuity, feet, HbA1c, lipid level and urine for albumin to creatinine ratio (ACR) and administration of vaccines.	
48	McDiarmid et al. 2001 [51]	On the schedule: NA	NA
		To the visit: Flashing reminder on check-in screen for patient to complete Diabetes Questionnaire and Reminder sheet (DQR). DQR directed patient attention to adherence to preventive care schedule and recommendations.	
		Patient information: DQR reminded providers to update diabetic flow chart and check feet. DQR directed patient attention to HbA1c, recent blood sugars, and self-management issues.	
49	McMahon et al. 2005 [78]	On the schedule: NA	NA
		To the visit: NA	
		Patient information: Intervention group received notebook computer, glucose and BP monitoring devices and access to care management website. Patients received educational resources, uploaded information from monitoring devices and could internal message the care manager using website.	
50	McMahon et al. 2012 [47]	On the schedule: NA	NA
		To the visit: NA	
		Patient information: Online care management (*I.1*): Patients are asked to upload glucose and blood pressure monitoring data and communicate securely with provider through patient portal; Telephone care management (*I.2*): Patients received phone calls bi-weekly to review glucose and blood pressure readings; Usual care with web training (*I.3*): Patients had access to online training materials that could be viewed at their discretion.	
51	Mehler et al. 2005 [79]	On the schedule: NA	Provider education
		To the visit: NA	
		Patient information: Providers urged to order lipid profiles for intervention groups by direct detailing or electronic detailing, reinforcing current lipid treatment guidelines and answering specific hyperlipidemia treatment questions.	
52	Meigs et al. 2003 [49]	On the schedule: NA	NA
		To the visit: NA	
		Patient information: Disease Management Application (DMA) enables decision support at time of patient contact, displays trended and tabular electronic laboratory data interactively linked to evidence-based treatment recommendations, aides workflow and links to additional patient and provider care resources.	
53	Meulepas et al. 2007 [30]	On the schedule: NA	NA
		To the visit: Diabetes Support Service (DSS) offered logistic support to providers and called up patients for laboratory testing (repeated 3-monthly and annual), foot examination, fundus photography and appointments with the dietician and diabetes nurse.	
		Patient information: Laboratory results sent to provider.	
54	Meulepas et al. 2008 [31]	On the schedule: NA	NA
		To the visit: DSS called patients for laboratory testing (repeated 3-monthly and annual), foot examination, fundus photography and appointments with the dietician and diabetes nurse.	
		Patient information: Practice nurse reviewed information and gave lifestyle advice to patient, traced risk factors and set short term goals with patient during quarterly visits.	
55	Moattari et al. 2013 [80]	On the schedule: NA	NA
		To the visit: NA	
		Patient information: Patients are asked to enter their self-monitored blood glucose level, kind and dose of insulin they used, and the amount and kind of daily food intake to the website every day. Healthcare team (physician, nurse, nutritionist) had access to patient’s files. The care team answers patients’ questions through the website and provides recommendations via email. Patients who need immediate response can ask questions using phone or SMS.	
56	Moorman et al. 2012 [81]	On the schedule: NA	NA
		To the visit: NA	
		Patient information: Patients were provided with a blank SMBG log at provider appointment in a pharmacist-run diabetic clinic and asked to return the completed log after two weeks via mail, fax, or telephone communication. Those patients in the post intervention cohort were sent reminder mailings one week before logs were due.	
57	Musacchio et al. 2011 [82]	On the schedule: NA	NA
		To the visit: NA	
		Patient information: Diabetologists, nurses and dietitians empower patient self-management, using patient clinic history in their EHR. Phone and internet utilized for patient communication.	
58	Nes et al. 2012 [83]	On the schedule: NA	NA
		To the visit: NA	
		Patient information: Patients were given access to web-based diaries housed on a secure server where they (1) registered their fasting blood glucose level in the morning, and eating behavior, medication compliance, exercise, and emotions three times per day; (2) received individualized situational feedback based on acceptance and commitment therapy; and (3) had access to mindfulness and relaxation exercises via audio file.	
59	Piette et al. 2000 [84]	On the schedule: NA	NA
		To the visit: NA	
		Patient information: Biweekly automated assessment calls to patients regarding: SMBG levels, symptoms, foot problems, chest pain, breathing problems, self-care problems. Nurse educator reviewed information and prioritize patients. Follow-up calls to discuss the reported problems, strategies for resolution, and education about importance of self-care, health monitoring, weight control, nutrition, and exercise.	
60	Rai et al. 2011 [18]	On the schedule: Automated outreach communication message to proactively motivate patients to schedule appointments.	NA
		To the visit: NA	
		Patient information: NA	
61	Ralston et al. 2009 [38]	On the schedule: NA	NA
		To the visit: NA	
		Patient information: Intervention group utilized web-based program to review online medical record, upload SMBG levels, create action plan, and exchange secure email with care manager. Care manager reviewed patient action plans, SMBGs and laboratory results at least 1×/week, adjusted hypoglycemic medications, guided patient health behavior, self-management support, and conferred with provider. Web program provided single-page summary of patient clinical diabetes information.	
62	Ryan et al. 2013 [85]	On the schedule: NA	Participants were given desktop computer, glucometer and test strips, Internet connection at home, periodic refresher training, and telephone user support.
		To the visit: NA	
		Patient information: Patients are asked to upload blood sugar levels and log into diabetes relationship management package. The web-based application provides educational material and motivational messages; access to providers for education, communication, and peer networking; chat with registered nurses. Nurses can view patients’ electronic medical records while chatting with patients.	
63	Sacco et al. 2009 [48]	On the schedule: NA	NA
		To the visit: NA	
		Patient information: Intervention group received weekly phone coaching for goal setting and self-management, SMBG testing, medication, nutrition, exercise, foot care, stress management, eye exam, dental care, and vaccinations.	
64	Sadur et al. 1999 [22]	On the schedule: Scheduled 2 hour cluster visits involving 10–18 patients every month for 6 mos.	NA
		To the visit: NA	
		Patient information: Intervention group received multidisciplinary care managed by diabetes nurse educator, two diabetologists, dietitian, behaviorist, and pharmacist. Nurse reviewed self-management by telephone twice monthly to every 3 days.	
65	Seto et al. 2012 [16]	On the schedule: Patients are contacted by telephone to schedule follow-up appointments.	Cost-benefit analysis of implementing and maintaining the registry
		To the visit: Reminder phone calls are made 24 h before the appointment. For the patients who did not return, the medical assistants called the patients or sent them a letter inquiring about access barriers.	
		Patient information: NA	
66	DM Smith et al. 1987 [27]	On the schedule: NA	Mailed educational booklet
		To the visit: Intervention group was mailed billfold-sized card with their provider and nurse name, clinic location, office hours, and telephone number, and single-page description on how to use card for appointments, medication refills and health problems with information of diabetic warning signs. Patient received a postcard reminder a week before each scheduled return visit. If patient missed an appointment intense follow-up by telephone and letter was implemented until another visit scheduled.	
		Patient information: NA	
67	KE Smith et al. 2004 [86]	On the schedule: Intervention group scheduled for baseline, 3 mos and 6 mos visits as routine care.	NA
		To the visit: NA	
		Patient information: Intervention group entered SMBG values, exercise logs, and communicated with provider via Web-based diabetes management application (MyCareTeam).	
68	Song et al. 2009 [87]	On the schedule: NA	NA
		To the visit: NA	
		Patient information: Intervention group utilized website with public space with diabetes self-management information, secure space to download SMBG values, calculator of daily caloric intake, physical activity log, stress measurement, feedback from specialist and FAQ area.	
69	Stone et al. 2012 [88]	On the schedule: NA	NA
		To the visit: NA	
		Patient information: (*I.1*) Active care management to lower intensity care coordination (ACM-to-CC), (*I.2*) Active care management to care coordination with continued home telemonitoring (ACM-to-CCHT), (*I.3*) Care coordination to continued care coordination (CC-to-CC), (*I.4*) Care coordination to usual care (CC-to-UC). Care coordination includes monthly educational phone calls, and home telemonitoring includes daily transmission of blood glucose, blood pressure, and weight.	
70	Subramanian et al. 2009 [23]	On the schedule: OA clinics offered same-day scheduling for patients.	NA
		To the visit: NA	
		Patient information: NA	
71	Tang et al. 2013 [89]	On the schedule: NA	NA
		To the visit: NA	
		Patient information: The interventions included: i) wireless upload of home glucometer readings to EHR, ii) comprehensive patient-specific diabetes summary status report which includes patient’s personalized action plan and treatment goals, diabetes complications risk, monitoring tests, medications, and health maintenance schedule, iii) nutrition and exercise logs, iv) insulin record; v) online messaging with the patient’s healthcare team, vi) nurse care manager and dietitian providing timely advice and medication management, and vii) personalized educational text and videos dispensed electronically by the care team. Primary care physicians were kept up to date about clinical changes through the shared EHR.	
72	Thomas et al. 2007 [26]	On the schedule: NA	NA
		To the visit: Letters recommending appropriate surveillance tests automatically sent quarterly to patient without HbA1c within 6 mos or LDL within 12 mos.	
		Patient information: Audit, feedback and patient reminder intervention utilized computerized diabetes registry to provide physicians with patient information.	
73	Tildesley et al. 2010 [90]	On the schedule: NA	NA
		To the visit: NA	
		Patient information: Intervention group uploaded SMBG levels every 2 weeks to web. Web-based system used to input medications, set alarms, view summary of SMBG levels, and send message to endocrinologist. Endocrinologist views data and, sends orders for insulin dosage and test frequency. Patients asked to perform laboratory test and visit endocrinologist every 3 mos.	
74	Weber et al. 2008 [53]	On the schedule: NA	Monetary incentive to providers for improvements in meeting evidence-based guidelines.
		To the visit: NA	
		Patient information: Assess ADA standards of care with EHR (EPIC). Intervention included health maintenance alerts to provider, best-practice-alerts, and nurse rooming tool.	
75	Yeh et al. 2006 [33]	On the schedule: NA	NA
		To the visit: Patient-Oriented education management system for diabetes using the Internet (POEM) sent reminders to intervention group 1 week before follow-up visit, HbA1c test period if more than 3 mos, and emergency calls for abnormal laboratory test results using emails and SMS.	
		Patient information: System automatically download patient’s records, prescriptions, laboratory test results, patient education materials and organizes into case folders based on patients’ medical service history from hospital for provider use at outpatient visit.	
76	Yoo et al. 2009 [91]	On the schedule: NA	NA
		To the visit: NA	
		Patient information: Phone reminder is used to remind patient to measure blood glucose and BP twice a day. Device attached to cellphone conducts glucose measurements and automatically sends the results to a central database. Automated messages of encouragement, reminders and recommendations are sent back to patients. SMS is used to receive exercise time and send information on healthy diet and exercise methods. Website is used to follow the blood glucose levels, blood pressure, and weight changes, and send individualized recommendations to patients when needed.	
77	Yoon and HS Kim 2008 [92]	On the schedule: NA	NA
		To the visit: NA	
		Patient information: Intervention group accessed website by cellular phone or wired internet sending SMBG values and drug information. Patient information automatically displayed on individual electronic chart on homepage. Patients could view recommendations from provider and laboratory test results. Recommendations sent to patient weekly, by SMS through cellular phone and wired internet.	

**Table 7:** Changes in other clinical outcomes

	Author	On schedule	To visit	With information	LDL (mg/dL)			SBP (mm/Hg)			Total cholesterol (mg/dL)			Triglycerides (mg/dL)			Other
					Int	Con	*p*	Int	Con	*p*	Int	Con	*p*	Int	Con	*p*	
39	Kirsh et al. 2007 [12]	✓			−16	−5.4	0.29	−14.8	−2.5	0.04							
70	Subramanian et al. 2009 [23]	✓			−6	−4	NS	1	−2	≤0.05							
5	Benhamou et al. 2007 [63]			✓													Glycaemia
6	Bond et al. 2006 [64]			✓													**BS**
7	Bond et al. 2007 [36]			✓				−6.8	−1	≤.010	−11.4	−5.1	≤.050				**HDL**
																	**Wt**
																	DBP
8	Carter et al. 2011 [37]			✓				−7	−8	NS							**BMI**
																	**Wt**
																	DBP
12	Cho et al. 2009 [66] (phone)			✓	NP	NA	NS				NP	NA	NS	NP	NA	NS	FPG
																	**2HPMG**
																	HDL
	Cho et al. 2009 [66] (internet)			✓	NP	NA	NS				NP	NA	NS	NP	NA	NS	FPG
																	**2HPMG**
																	HDL
13	Cho et al. 2011 [67]			✓							−0.2^c^	NA	.043	−0.5^c^	NA	NS	FBG
																	HDL
											NA	0.1^c^	NS	NA	−0.3^c^	NS	
																	Liver enzymes
18	Dijkstra et al. 2005 [54]			✓				1.1	−0.2	NS	-0.2^c^	−0.2^c^	NS				**DBP**
																	CR
24	Glasgow et al. 2003 [70] (overall)			✓	−8.3	NA	≤.010				−11.7	NA	≤.010	−16.5	NA	≤.001	**Lipid ratio**
																	HDL
	Glasgow et al. 2003 [70] (peer support)			✓													Lipid ratio
	Glasgow et al. 2003 [70] (tailored self-management)			✓													Lipid ratio
26	Grant et al. 2008 [55]			✓	NP	NP	NS										Blood pressure
27	Harno et al. 2006 [71]			✓	−0.18^c^	0.11^c^	NP	1	1	NP	−0.21^c^	0.12^c^	NP	−0.05^c^	0.21^c^	NP	**DBP**
																	**FBG**
																	BMI
																	HDL
																	CR
32	HS Kim et al. 2005 [44] “Effects of an Internet-based Intervention…”			✓							−13.50	NA	NS	27.30	NA	NS	**FPG**
																	**2HPMG**
																	HDL
34	HS Kim 2007 [39] “A randomized controlled trial of a nurse short-message service…”			✓													**2HPMG**
																	FPG
35	HS Kim 2007 [40] “Impact of web-based nurse’s education…”			✓													**FPG** ^**d**^
																	**2HPMG**
36	HS Kim and H. Jeong 2007 [41] “A nurse short message service by cellular phone…”			✓													**2HPMG**
																	FPG
37	HS Kim and M. Song 2008 [43] “Technological intervention for obese patients with type 2 diabetes”			✓							−5.1	9.9	0.04	4.5	40.8	0.62	**FPG**
																	**2HPMG**
																	HDL
38	SI Kim and HS Kim 2008 [73] “Effectiveness of mobile and internet intervention…”			✓													**2HPMG**
																	FPG
39	Kwon et al. 2004a [74]			✓							−0.9	NA	NS	−24.4	NA	≤.007	**HDL**
																	FPG
40	Kwon et al. 2004b [45]			✓	−1.93	NA	NS				−3.33	NA	NS	−19.5	NA	NS	HDL
					NA	1.88	NS				NA	7.3	NS	NA	13.5	NS	FBG
49	McMahon et al. 2005 [78]			✓	−6	−5	NS	−10	−7	≤0.01				−38	NA	≤0.01	DBP
														NA	−2	NS	**HDL**
50	McMahon et al. 2012 [47] (online care)			✓	−4.0	NA	0.29	−0.3	NA	0.89	−7.8	NA	0.07	−25.5	NA	0.01	DBP
																	HDL
																	Wt
																	BMI
	McMahon et al. 2012 [47] (telephone care)			✓	−5.5	NA	0.12	−6.7	NA	.006	−8.5	NA	0.05	−6.5	NA	0.68	**DBP**
																	**HDL**
																	Wt
																	BMI
	McMahon et al. 2012 [47] (usual care with web training)			✓	5.8	NA	0.12	−3.1	NA	0.30	−10.7	NA	0.02	−26.4	NA	0.08	**DBP**
																	HDL
																	Wt
																	BMI
52	Meigs et al. 2003 [49]			✓	−14.7	−9.4	0.30	0.8	−2.2	0.03							DBP
55	Moattari et al. 2013 [80]			✓	−8.2	5.1	<0.02				3.9	−0.8	0.69	45.3	−7.9	0.34	HDL
																	FBS
61	Ralston et al. 2009 [38]			✓				NP	NP	NS	NP	NP	NS				DBP
62	Ryan et al. 2013 [85]			✓	−15.7	NA	0.10	4.4	NA	0.30	−15.5	NA	0.14	21	NA	0.01	DBP
																	HDL
																	BMI
67	KE Smith et al. 2004 [86]			✓	−18	NA	NS	−6	NA	NS	−25	NA	NS	−9	NA	NS	**BMI**
																	DBP
																	HDL
68	Song et al. 2009 [87]			✓													FBG
71	Tang et al. 2013 [89]			✓	−6.1	0.0	.001	−7.1	−5.3	NS							Wt
																	DBP
																	Framingham cardiovascular risk
73	Tildesley et al. 2010 [90]			✓							NP	NP	S	NP	NP	S	
76	Yoo et al. 2009 [91] “A ubiquitous chronic disease care system using cellular phones and the internet”			✓	−0.4^c^	−0.1^c^	0.03				−0.5^c^	0.0^c^	0.01				**Wt**
																	**BMI**
					−0.4^c^	NA	≤.001	−7	NA	≤.001	−0.5^c^	NA	≤.001	−0.22^c^	NA	≤.003	
																	**DBP**
																	**HDL**
					NA	−0.1^c^	NS	NA	−4.00	NS	NA	0.0^c^	NS	NA	0.08^c^	NS	
77	Yoon and HS Kim 2008 [92]			✓							−3.1^a^	29.3^a^	0.18	−31.1^a^	52.3^a^	0.28	**2HPMG**
																	HDL
																	FPG
4	Bailie et al. 2004 [62]	✓		✓				66^e^	NA	NS							DBP
								0^a^	NA	NS							
11	Cho et al. 2006 [57]	✓		✓							−0.14^ac^	−0.31^ac^	NS	−0.08^ac^	0.4^ac^	≤.050	HDL
																	FBG
																	CR
16	de Grauw et al. 2002 [19]	✓		✓				−3	NA	0.20	−31	NA	≤.001	−41	NA	≤.001	HDL
																	FBG
																	**DBP**
30	Jones and Curry 2006 [50]	✓		✓	−2.5	−5.3	NS	−1.1	−0.7	NS							DBP
					−2.5	NA	NS	−1.1	NA	NS							
																	Wt
					NA	−5.3	0.03	NA	−0.7	NS							
45	MacLean et al. 2009 [20]	✓		✓	−12.5	−13.6	NS										BMI
																	DBP
28	Holbrook et al. 2009 [28]		✓	✓	−0.05^c^	0.02^c^	NS	−4.7	0.3	0.04							**DBP**
																	BMI
																	Alb
29	Hurwitz et al. 1993 [72]		✓	✓													RPG
53	Meulepas et al. 2007 [30]		✓	✓				−5	4	≤.050	−0.4^c^	−0.3^c^	NS	−0.1^c^	0.1^c^	NS	**FBG**
																	DBP
54	Meulepas et al. 2008 [31]		✓	✓				4	5	NS	−0.3^c^	−0.3^c^	NS	−0.3^c^	−0.3^c^	NS	FBG
																	BMI
																	DBP
72	Thomas et al. 2007 [26]		✓	✓	−4.9	−4	0.61	−0.5	NA	NS							DBP
								NA	1.7	NS							
75	Yeh et al. 2006 [33]		✓	✓							−24	−22	≤.050				**FBG**
19	Edelman et al. 2010 [34]	✓	✓	✓				−13.7^a^	−6.4^a^	0.01							**DBP**
42	Lin et al. 2007 [29]	✓	✓	✓	−0.59^c^	NA	<0.01	−4.7	NA	NS							DBP
																	Wt
					NA	−0.16^c^	NS	NA	1	NS							

*Int* intervention, *Con* Control, *NA* not Applicable, *NS* non-significant (p-value>0.05), *S* significant (p-value≤0.05), *NP* not provided

Results are differences in mean before and after implementation of intervention except those indicated with the following superscripts

^a^Multiple measurements are presented over time after the intervention in the paper, but the last measurement is used to calculate the difference in this table

^b^Multiple measurements are presented over time after the intervention in the paper, but median change is reported

^c^Unit is mmol/l

^d^HbA1c<7 at baseline

^e^% with BP < 140/90

Other outcome measures include: *DBP* Diastolic blood pressure, *CR* Creatinine, *Wt* Weight, *FPG* Fasting plasma glucose, *FBG* Fasting blood glucose, *FBS* Fasting blood sugar, *BS* Blood sugar, *2HPMG* 2-hour post-meal glucose, *FBG* Fasting blood glucose, *RPG* Random plasma glucose, *Alb* Albuminuria

Bolded text in ‘Other’ category indicates significant finding

**Table 8:** Changes in behavioral outcomes

	Author	On schedule	To visit	With information	Improved self-management								Increased outpatient care services^a^				Decreased acute care utilization		Improved adherence to ADA guidelines			Other
					SMBG testing	Self-efficacy score	QOL score	Patient satisfaction	Physical activity exercise	Foot care	Diet	Nutrition	Lab tests completed	Vaccination	Provider visit	Specialist visit	Emergency visits	Hospital admissions	Foot exam	Eye exam	Processes of care	
1	Anderson et al. 2003 [15]	✓																		**S**		
41	Lafata et al. 2002 [14]	✓											**A1c**		**S**					**S**		
													LDL									
60	Rai et al. 2011 [18]	✓											**A1c**		**S**							
70	Subramanian et al. 2009 [23]	✓											**A1c↓**		NS		NS	NS				
													LDL									
													**Alb↓**									
2	Austin and Wolfe 2011 [24]		✓										**A1c**									
													LDL									
17	Derose et al. 2009 [25]		✓										**A1c**									
													**LDL**									
													**Alb**									
66	DM Smith et al. 1987 [27]		✓												**S**			NS				**Kept scheduled visits↑**
																						**Scheduled appointments↑**
																						Missed appointments
																						Medication refills
3	Avdal et al. 2011 [61]			✓											**S**							
5	Benhamou et al. 2007 [63]			✓	NS		**S**															
8	Carter et al. 2011 [37]			✓					NS		NS											**Diabetes knowledge↑**
																						**Diabetes management practice↑**
																						**Perceived physical health status↑**
																						**Perceived mental health status↑**
10	Cherry et al. 2002 [46]			✓			S	NS							**S↓**		NS	NS				Patients who feel more connected
																						Medication compliance
																						Number of post-discharge visits
12	Cho et al. 2009 [66] (internet vs. phone)			✓	NS^b^			NS^a^														
13	Cho et al. 2011 [67]			✓	**S**																	
15	Ciemins et al. 2009 [52]			✓									A1c						S	S	S	
													**LDL**									
													**Lip**									
													**AlbCR**									
18	Dijkstra et al. 2005 [54]			✓									A1c			NS			S	NS		Urine exam < 12 mo.
													TC									Weight checked
													BP									BMI checked
																						**Physical exercise advised↑**
																						**Smoking discussed↑**
													CR									
24	Glasgow et al. 2003 (overall) [70]			✓					NS		S										**S**	**Psychosocial outcomes (total support scale)↑**
	Glasgow et al. 2003 (peer support) [70]			✓					NS		NS										NS	**Psychosocial outcomes (total support scale)↑**
	Glasgow et al. 2003 (tailored self-management) [70]			✓					NS		S										NS	**Psychosocial outcomes (total support scale)**
25	Glasgow et al. 2004 [58]			✓	**S**		NS	S				S	**BP**						S	S		**Self-management goal setting↑**
													**Alb**									Depressive symptoms
32	HS Kim et al. 2005 [44]			✓				**S**														
33	HS Kim et al. 2006 [42]			✓					**S**	**S**	NS											**Medication taking↑**
40	Kwon et al. 2004 [45]			✓				A														
43	Litzelman et al. 1993 [75]			✓						**S**									**S**			
44	Lorig et al. 2010 [76]			✓		**S**			NS						NS							**Health distress↓**
																						Depression
																						Patient activation
46	McCarrier et al. 2009 [77]			✓		**S**																Program usage
50	McMahon et al. 2012 [47] (online care)			✓																		Diabetes distress
	McMahon et al. 2012 [47] (telephone care)			✓																		**Diabetes distress**
	McMahon et al. 2012 [47] (usual care with web training)			✓																		**Diabetes distress**
51	Mehler et al. 2005 [79]			✓									**LDL**									
													Lip									
52	Meigs et al. 2003 [49]			✓									**A1c**						S	NS		
													**LDL**									
56	Moorman et al. 2012 [81]			✓	NS																	Follow up appointment kept
																						**Returned SMBG log**
57	Musacchio et al. 2011 [82]			✓												**S↓**						
58	Nes et al. 2012 [83]			✓			NP															Diabetes distress
59	Piette et al. 2000 [84]			✓		S	NS	**S**														**Depression↓**
																						**Days in bed because of illness↓**
																						Anxiety
62	Ryan et al. 2013 [85]			✓			NS															
63	Sacco et al. 2009 [48]			✓		**S**			**S**	**S**	**S**											
68	Song et al. 2009 [87]			✓																		**Diabetes care knowledge↑**
																						**Diabetes care behavior↑**
71	Tang et al. 2013 [89]			✓				**S**							NS							Diabetes distress
74	Weber et al. 2008 [53]			✓									**A1c**	**In**							S	Percentage documented non-smoker
														**Pn**								
													**LDL**									
													**Alb**									
76	Yoo et al. 2009 [91]			✓	NP																	
65	Seto et al. 2012 [16]	✓	✓										A1c									Appointment adherence
4	Bailie et al. 2004 [62]	✓		✓									**A1c**	In					**S**	**S**		**Weight measurement↓**
													CR	Pn								
																						BMI measurement
													AlbCR									
																						Counseling about diet, activity, weight, smoking, alcohol, medication
													**Lip**									
14	Chumbler et al. 2005 [21]	✓		✓											**S**	NS	**S**	**S**	NS	NS		
16	de Grauw et al. 2002 [19]	✓		✓									**A1c**		**S**	**S**						
													**SBP**									
													**DBP**									
20	Edwards et al. 2012 [17]	✓		✓									**A1c**	**In**					**S**	**S**	**S**	
													**Lip**	**Pn**								
22	Fischer et al. 2011 [13] (mailed patient report card)	✓		✓									A1c									
													BP									
													LDL									
30	Jones and Curry 2006 [50]	✓		✓										In					S	S		
														Pn								
45	MacLean et al. 2009 [20]	✓		✓			NS		**S**	NS	NS		A1c		**S↓**	**S↓**	S	S				Functional status
																						Blood testing
													**LDL**									
																						**Cost↓**
													**CR**									
64	Sadur et al. 1999 [22]	✓		✓	NS	NS		NS	NS	NS		**S**			NS			**S**				
23	Fischer et al. 2012 [69]		✓	✓	**S**			A														No-shows
																						Cancellations
28	Holbrook et al. 2009 [28]		✓	✓					NS				**A1c**		**S**				**S**		**S**	
													**BP**									
													**LDL**									
													**Alb**									
47	McDermott et al. 2001 [32]		✓	✓									A1c	In				**S**	**S**	**S**		Hypertension receiving treatment,
													**BP**	Pn								
																						Retinopathy noted,
																						**Dyslipidemia noted↑,**
																						Self monitoring,
																						**Urinary ACR checked <12 mo.↑,**
																						**Weight checked↑,**
																						**BP checked ↓,**
													**Lip**									Albuminuria on ACE inhibitor treatment
													**CR**									
48	McDiarmid et al. 2001 [51]		✓	✓									**A1c**						**S**	**S**		**Office visit with inquiry of hypoglycemia↑**
													LDL									
													Alb									
53	Meulepas et al. 2007 [30]		✓	✓									**A1c**						**S**	**S**		**Smoking status checked↑,**
													**BP**									
																						**BMI checked↑**
													**FBG**									
													DBP									
													TC									
													CR									
54	Meulepas et al. 2008 [31]		✓	✓					**S**													Percentage of non-smokers,
																						BMI checked
72	Thomas et al. 2007 [26]		✓	✓									**A1c**									
													**LDL**									
19	Edelman et al. 2010 [34]	✓	✓	✓											**S↓**		**S**	NS				**Lightheadedness or falls↓,**
																						**Medication adherence**

***S*** significant (p-value≤0.05), *NS* non-significant (p-value>0.05), ↓ decrease, ↑ increase

^a^If outpatient care services are significantly reduced (not increased) after intervention, it is represented using ↓

*A1c* Hemoglobin HbA1c test, *SBP* Systolic blood pressure, *DBP* Diastolic blood pressure, *BP* Blood pressure, *CR* Creatinine, *TC* Total cholesterol, *FBG* Fasting blood glucose, *Alb* Urine microalbumin, *AlbCR* Microalbumin/creatinine, *BMI* Body mass index, *Lip* Lipid profile, *In* Influenza vaccination, *Pn* Pneumonia vaccination

Bolded text indicates significant findings

^b^Difference between groups, *A* Measured only after the intervention, *NP* p-value is not given
